# Senescent cells and the incidence of age‐related diseases

**DOI:** 10.1111/acel.13314

**Published:** 2021-02-08

**Authors:** Itay Katzir, Miri Adler, Omer Karin, Netta Mendelsohn‐Cohen, Avi Mayo, Uri Alon

**Affiliations:** ^1^ Department of Molecular Cell Biology Weizmann Institute of Science Rehovot Israel; ^2^ Broad Institute of Massachusetts Institute of Technology and Harvard Cambridge MA USA; ^3^ Department of Computer Science Weizmann Institute of Science Rehovot Israel

**Keywords:** age‐related disease, aging, cancer, cellular senescence, diabetes, electronic medical records, fibrosis, incidence rate, idiopathic pulmonary fibrosis, mathematical model, osteoarthritis

## Abstract

Age‐related diseases such as cancer, cardiovascular disease, kidney failure, and osteoarthritis have universal features: Their incidence rises exponentially with age with a slope of 6–8% per year and decreases at very old ages. There is no conceptual model which explains these features in so many diverse diseases in terms of a single shared biological factor. Here, we develop such a model, and test it using a nationwide medical record dataset on the incidence of nearly 1000 diseases over 50 million life‐years, which we provide as a resource. The model explains incidence using the accumulation of senescent cells, damaged cells that cause inflammation and reduce regeneration, whose level rise stochastically with age. The exponential rise and late drop in incidence are captured by two parameters for each disease: the susceptible fraction of the population and the threshold concentration of senescent cells that causes disease onset. We propose a physiological mechanism for the threshold concentration for several disease classes, including an etiology for diseases of unknown origin such as idiopathic pulmonary fibrosis and osteoarthritis. The model can be used to design optimal treatments that remove senescent cells, suggeting that treatment starting at old age can sharply reduce the incidence of all age‐related diseases, and thus increase the healthspan.

## Graphical abstract

The incidence of age‐related diseases rises exponentially and drops at very old ages, but the reason is unclear. We explain this with a model in which disease onset occurs when senescent‐cell stochastic accumulation crosses a threshold, with only a fraction of individuals susceptible. The model describes new, extensive medical‐record incidence data with only two parameters per disease, and suggests an etiology for diseases of unknown origin such as idiopathic pulmonary fibrosis and osteoarthritis.

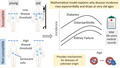

## 1 INTRODUCTION

Age‐related diseases are the main causes of death and disability (Austad, 2016; Gladyshev & Gladyshev, 2016; Olshansky et al., 2007). These diseases include cardiovascular disease, cancer, Alzheimer's disease, diabetes, kidney failure, and osteoarthritis. They affect different organ systems and have different origins, including mutations, dysregulated homeostasis, fibrosis, and degenerative processes.

**FIGURE 1:**
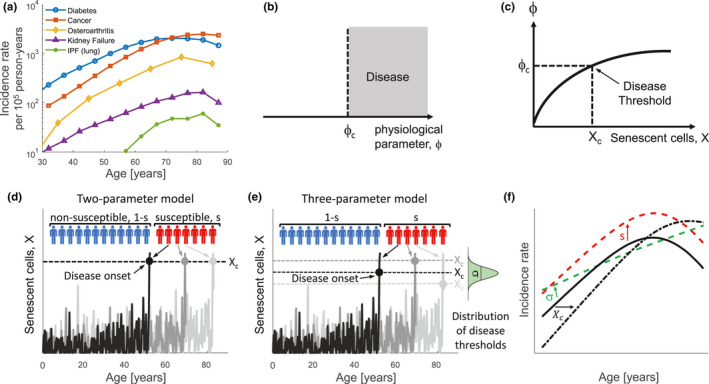
Diseases caused by threshold‐crossing of a parameter affected by senescent cells are predicted to have an exponential incidence curve with a decline at old ages. (a) Incidence curves for several age‐related diseases, from (Public Health Agency of Canada, 2011; National Cancer Institute et al., 2018; Navaratnam et al., 2011; Oliveria et al., 1995). (b) We assume that disease onset occurs when a physiological parameter *ϕ* exceeds a threshold, *ϕ*_c_. (c) *ϕ* is a rising function of senescent‐cell level, X, so that *ϕ*_c_ is crossed when X exceed a disease threshold $X_{c}$. (d) The senescent‐cell levels of three susceptible individuals simulated by the SR model. The disease arises as a first‐passage‐time process when X crosses $X_{c}$. (e) In the three‐parameter model, the threshold $X_{c}$ for each person in the susceptible fraction of the population is drawn from a Gaussian distribution with mean $\bar{X_{c}}$ and standard deviation σ. (f) Effect of the model parameters on the incidence curve. The parameters are $X_{c}=14,\;s=0.05$ (black), $X_{c}=14,\;s=0.15$ (dashed red), $X_{c}=16,\;s=0.05$ (dashed black), $\bar{\mathrm{Xc}}=14,\;s=0.05,\;\sigma =3$ (dashed green)

Despite the differences between these pathologies, they have certain universal features in terms of their incidence rate. The incidence rate of a disease is defined as the number of new cases per year divided by the size of the population (Rothman, 2012). The incidence rate of each age‐related disease rises roughly exponentially with age (Belikov, 2019; Zenin et al., 2019). For many of the diseases, the incidence rate then drops at very old ages. Interestingly, the slope of the rising part of the incidence curve is similar for many age‐related diseases, in the range of 6–8% per year (Belikov, 2019; Zenin et al., 2019) (Figure 1a). This similarity hints at a common biological process of aging that governs the onset of these different diseases (Finch & Kirkwood, 2000; Franceschi et al., 2018; Justice et al., 2018; Kaeberlein, 2017; Kennedy et al., 2014; Kirkland, 2016; Kirkwood, 2005; Kritchevsky & Justice, 2020; Olshansky et al., 2007). It is thus of interest to develop theories for the origin of the incidence of age‐related diseases, in order to detect such a common process.

To explain the incidence curves, theoretical work has focused on specific classes of diseases, primarily on cancer. An early theory called the multiple‐hit theory (Armitage & Doll, 1954; Nordling, 1953), noted that cancer often depends on several mutations in the same cell. The probability that a cell acquires all these mutations rises with age as a power law. Thus, according to this model, the incidence of cancer *I(t)* goes as $I∼At^{\gamma }$. The multiple‐hit model has two parameters, an amplitude A and an effective mutation number γ. It provides reasonable fits to the incidence curves of many cancers (Armitage & Doll, 1954; Nordling, 1953). However, it cannot explain cancers based on a single mutational event, such as chronic myeloid leukemia (CML) that depends on a single translocation (Druker et al., 2001), which also show an exponentially rising incidence with age (Palmer et al., 2018). It also does not explain the slowdown or drop in incidence at very old ages. This drop is usually explained in epidemiology as due to population heterogeneity (e.g., certain people are at a lower risk to begin with) or cohort effects (Burch, 1965; Hanson et al., 2015; Hawkes et al., 2012; Horiuchi & Wilmoth, 1998). Other theories suggest that the drop may arise from a slowdown in stem cell divisions leading to fewer mutational events at very old ages and thus lower cancer incidence (Tomasetti et al., 2019).

A more recent theory for the age‐related incidence of cancer and infectious diseases is based on the hypothesis that impairment of the adaptive immune system with age causes the observed exponential increase of incidence rate (Palmer et al., 2018). Palmer et al. assume that the rate of decline of the thymus with age is the main temporal process that drives incidence. The thymus is the source of T‐cells that remove cancer cells and infected cells and is thought to decline exponentially with age. Palmer et al. model the growth and removal of cancer cells and estimate the probability to reach a critical number of cancer cells, in which the cancer can build a microenvironment that avoids further removal. The incidence rate of cancer in this model has three parameters, $I∼A/(e^{e^{‐\alpha \left(t‐\tau \right)}}‐1)$, where the thymus decay rate is $\alpha =0.044{[year}^{‐1}],$ the amplitude is A, and τ is the “pivot age” which marks a transition from lower to higher risk. This model, called IMII, describes the incidence curves of many cancers and infection reasonably well. Like the multiple‐hit model, this model does not attempt to explain the drop in incidence at very old ages.

The existing explanations for the incidence of age‐related diseases seem to focus primarily on cancer. They do not apply to other classes of diseases such as fibrotic and metabolic diseases, in which the role of adaptive immunity or mutational hits are thought to be less central. Some age‐related diseases do not currently have a clear mechanism for their origin, such as idiopathic pulmonary fibrosis (IPF). Thus, it is of interest to develop a theory that can explain the incidence of diverse classes of age‐related diseases based on a shared biological process (Santra et al., 2019).

Here, we develop such a theory, based on a process which has been shown in recent years to be causal for a wide range of age‐related pathologies: the accumulation of *senescent cells* (Baker et al., 2016; Kirkland, 2016; Xu et al., 2018). Senescent cells are damaged cells that stop dividing and accumulate in the body with age. They secrete factors, collectively known as SASP (Senescence Associated Secretion Profile) (Basisty et al., 2020; Coppé et al., 2010; Tchkonia et al., 2013), which cause inflammation and reduce progenitor cell division. Removing senescent cells from mice extends life span and ameliorates many age‐related diseases (McHugh & Gil, 2018; Short et al., 2019) including cancer (Short et al., 2019), Alzheimer's disease (Zhang et al., 2019), osteoporosis (Kim et al., 2017), renal dysfunction (Baker et al., 2016), cardiovascular disease (Childs et al., 2016; McHugh & Gil, 2018), metabolic diseases (Palmer et al., 2015), idiopathic pulmonary fibrosis (Schafer et al., 2017a), and osteoarthritis (Jeon et al., 2017).

Recent work by Karin et al. (Karin et al., 2019; Karin & Alon, 2020) studied senescent‐cell dynamics with age, and used these dynamics to explain the distribution of death times in mice and humans. Karin et al. showed that senescent cells are produced and removed with a half‐life of days in young mice, but their removal rate slows down in old mice to a half‐life of weeks. These data, together with longitudinal measurement of senescent cells in mice (Burd et al., 2013), were used to develop a stochastic model for senescent‐cell production and removal, called the saturated‐removal (SR) model. The SR model shows that senescent cells slow their own removal rate, which leads to wide variations between individuals in the number of senescent cells at old ages. Assuming that death occurs when senescent cells exceed a threshold (following Sacher (Sacher, 1956)), Karin et al showed that the SR model explains the distribution of times of death. To do so, they computed the distribution of the first‐passage‐time of senescent cells across the threshold. This provides the well‐known Gompertz law (Olshansky et al., 2007), in which risk of death rises exponentially with age and slows at very old ages.

Since senescent cells are implicated in many age‐related diseases, and since a threshold‐crossing event of senescent cells in the SR model has an exponentially rising probability with age, we asked whether age‐related diseases can be modeled as a threshold‐crossing phenomenon in which senescent cells exceed a disease‐specific threshold (Belikov, 2019). To explain the drop in incidence at very old ages, we add to this model the epidemiological notion of heterogeneity (Burch, 1965; Hanson et al., 2015; Hawkes et al., 2012; Horiuchi & Wilmoth, 1998), in which some people are more susceptible to the disease than others. We show that the SR model with differential susceptibility provides a model with 2 or 3 free parameters that can explain a wide range of age‐related incidence curves. This includes the incidence of many types of cancer, major fibrotic diseases, and hundreds of other age‐related disease states obtained from a large‐scale medical record database with 50 million person‐years (Balicer & Afek, 2017), as well as from UKbiobank. We provide specific biological interpretations for the threshold mechanism for classes of disease, providing putative etiologies for diseases with unknown origin, such as IPF and osteoarthritis.

This conceptual picture explains why different diseases have similar exponential rise in incidence and a drop at very old ages, based on a shared biological process, the accumulation of senescent cells. It also can be used to optimize the frequency of treatments that eliminate senescent cells, showing that even infrequent treatment starting at old age can reduce the incidence of a wide range of diseases.

## 2 RESULTS

### 2.1 Diseases caused by threshold‐crossing of a parameter affected by senescent cells are predicted to have an exponential incidence curve with a decline at old ages

In this section, we present a general mechanism that can lead to the observed incidence curves. The next sections provide examples of classes of diseases, which show the hallmarks of this general mechanism.

For clarity, we begin by spelling out the model and then describe the reasoning behind it. The model has two versions, a two‐parameter and a three‐parameter version. In the two‐parameter model, each disease has two parameters: the fraction of the population that is susceptible to the disease *s*, and the disease threshold $X_{c}$. For each individual, one simulates senescent‐cell abundance using the SR model. If the individual is not susceptible (probability 1 − *s*), the disease does not occur. If the individual is susceptible (probability *s*), disease onset occurs when the senescent‐cell abundance first crosses the threshold, ${X\left(t\right)>X}_{c}$. Thus, each disease is characterized by two parameters, *s* and $X_{c}$.

The three‐parameter version posits a distribution of disease thresholds instead of a single threshold $X_{c}$. The disease threshold for each susceptible individual is drawn from a normal distribution with mean $X_{c}$ and standard division σ. Thus, each disease is characterized by three parameters: $X_{c}$, *s*, and σ.

The reasoning for the model is as follows. We will show that a disease has an approximately exponential incidence curve with age, which declines at very old ages, in the following situation:

- Onset of the disease occurs when a physiological parameter *ϕ* exceeds a threshold, *ϕ*_c_ (Figure 1b).
- Senescent cells are a causal factor for the disease: The parameter *ϕ* increases due to the total body senescent‐cell level X. Increasing levels of X can thus cause *ϕ* to exceed its threshold *ϕ*_c_. The threshold is crossed when X reaches a level $X_{c}$, called the disease threshold (Figure 1c).
- The disease threshold $X_{c}$ varies between people due to genetics and environment.

Total body senescent‐cell level *X* can affect *ϕ* in several ways (point ii above). They secrete SASP into the circulation which sends inflammatory signals and negatively impacts stem‐cell proliferation (Chang et al., 2016; Yosef et al., 2016). High senescent‐cell levels may also saturate or exhaust the immune cells that remove them, reducing total body immune capacity. Senescent cells also have local effects in each organ. We discuss specific mechanisms below for selected diseases.

When the above conditions are met, the disease arises in a given person when senescent‐cell level X crosses the threshold $X_{c}$ (Figure 1d). Thus, incidence (onset event) of the disease can be described as a first‐passage‐time problem, asking when the stochastic process of senescent‐cell accumulation first crosses the threshold $X_{c}$. It is likely that X must exceed the threshold for sufficient time for the disease to be expressed symptomatically. In practice, once X crosses the threshold, it tends to remain above the threshold for extended periods of time (SI section 1). Thus, a first‐passage‐time problem is a reasonable approximation for disease onset.

We assume that the SR model of Karin et al gives the dynamics ofX. In this model, X is governed by a stochastic differential equation$:dX/dt=\eta t‐\frac{\beta X}{\kappa +X}+\sqrt{2\epsilon }\xi$, with a production rate that rises with age ηt, a saturating removal rate $\frac{\beta X}{\kappa +X}$, and noise modeled as a white‐noise term $\sqrt{2\epsilon }\xi$. Model parameters for humans were provided in Karin et al (parameters given in SI section 2, we assume that all individuals have the same parameters). Simulations show stochastically rising trajectories of senescent cells, X (Figure 1d).

Karin et al also solved the first‐passage‐time problem, the distribution of times in which Xfirst crosses a threshold. The solution is an exponential incidence curve that slows at very old ages. The probability of crossing the threshold $X_{c}$ rises exponentially with age, $e^{\alpha t}$, with a slope of approximately $\alpha \approx \frac{\eta X_{c}}{\epsilon }$, where η and ϵ are the senescent‐cell production and noise parameters. This explains the exponential rise of incidence. Each threshold $X_{c}$ provides a different exponential slope. The threshold for death in Karin et al was estimated to be $X_{c}=X_{\mathrm{death}}=17$ (the units are such that X in young individuals is X=1, see (Karin et al., 2019)). Here, we model different disease thresholds as values of $X_{c}$ which do not exceed $X_{\mathrm{death}}$.

The disease threshold explains the exponential rise of incidence but does not provide the decline at very old ages. To explain the decline of incidence at old ages, we add the notion of population heterogeneity from epidemiology (Burch, 1965; Hanson et al., 2015; Hawkes et al., 2012; Horiuchi & Wilmoth, 1998). The idea is that people differ in their risk for a given disease. To model this, we assume that only a fraction *s* of the population has a low disease threshold, due to genetic and environmental factors. We call this the *susceptible* fraction (Figure 1d,e). The remaining population has high values of the disease threshold that are not reached during normal aging. We call these the *non*‐*susceptible* fraction of the population. Thus, at very old ages, most of those that are susceptible have already succumbed to the disease. At these ages, the population is dominated by the non‐susceptible fraction. This results in a decline in incidence rate (Figure 1f).

The simplest version of the model therefore has two free parameters for each disease: the susceptible fraction s and the disease threshold $X_{c}$ (Figure 1d). The parameters of the SR model for the senescent cells stochastic process are considered to be fixed and are taken from ref (Karin et al., 2019). Analytical formula for the incidence as a function of $X_{c}$ and s are provided in Methods (Equation 1). Software for determining these parameters from incidence data is also provided (Methods).

A slightly more elaborate model assumes that the threshold $X_{c}$varies from person to person within the susceptible population. We use a simple three‐parameter version that assumes a Gaussian distribution of disease thresholds with mean $\bar{X_{c}}$ and standard deviation σ (Figure 1e). Thus, the three parameters are s, $\bar{X_{c}}$ and σ (incidence is given by Equation 2 in Methods).

The effects of the parameters on the incidence curve are shown in Figure 1f. Increasing susceptibility s raises the incidence curve, because more people get the disease. Increasing $X_{c}$ shifts incidence to older ages because it takes longer for senescent cells level to cross the disease threshold. It also shifts the age of maximal incidence to older ages. In the three‐parameter model, increasing σ decreases the slope of the incidence curve since it allows low threshold values $X_{c}$ that can be reached at younger ages.

### 2.2 The model describes well the incidence of age‐related diseases from a nationwide medical database

To test the model requires comprehensive incidence data. To obtain such a global view of age‐related diseases, we provide and analyze incidence data from a large medical record database from Clalit health services (Balicer & Afek, 2017). This dataset includes about half of the Israeli population over a period of 14 years (2005–2018) totaling about 50 million life‐years, with broad socioeconomic and ethnic representation. We analyzed disease category codes (ICD9, level two codes) found in the records of at least 10^4^ people, totaling 877 disease category codes (See SI section 3). To define age‐related diseases, we computed the average slope of the incidence curve in the age range of 30–80 years. We find 375 codes for female and 444 codes for male that are at least mildly age‐related, defined by an average incidence slope of more than 3% per year. These include 165 codes for female and 232 codes for male that are strongly age‐related, defined by incidence slope of more than 7% per year.

**FIGURE 2:**
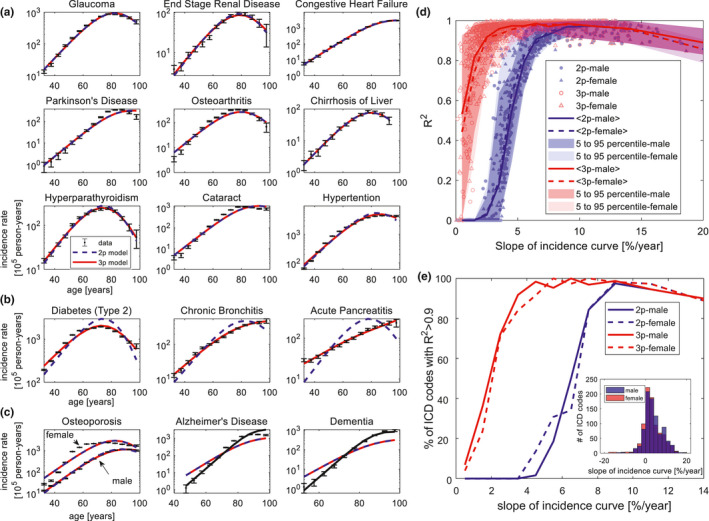
The model describes the incidence curves of a wide range of age‐related diseases. (a) The two‐parameter (2p) and three‐parameter (3p) models fit the incidence curves of many age‐related diseases. Data from Clalit ICD9 codes for females (similar results for males in Figure S2). (b) Examples where the three‐parameter model provides an excellent fit, but not the two‐parameter model. (c) The model does not describe well the incidence of osteoporosis in women (left panel). It cannot capture the incidence curve of Alzheimer disease and dementia using the maximal value of $X_{c}=X_{\mathrm{death}}=17$; the fit is improved with $X_{c}\approx 20$ for dementia and $X_{c}\approx 23$ for Alzheimer's disease (black line). (d) Coefficient of determination *R* ^2^ for fits of the two‐parameter (2p) and three‐parameter (3p) models to incidence of ICD9 codes as a function of mean slope of incidence between ages 30 and 80. (e) Percent of ICD9 codes with *R* ^2^ > 0.9 as a function of slope. Inset: number of ICD9 codes as a function of slope. Error bars are 95% CI

The two‐parameter model describes well the strongly age‐related ICD9 codes: 90% of the codes show *R* ^2^ > 0.9 (<*R* ^2^> = 0.95, median *R* ^2^ = 0.97, Figure 2d,e). The typical disease threshold values $X_{c}$ range between 12 and 16 (compared with X levels of about 1 in young individuals). These diseases include some of the most common age‐related conditions such as Parkinson's disease, glaucoma, congestive heart failure, end‐stage renal disease, liver cirrhosis, cataract, hypertension, and osteoarthritis (Figure 2a, Figure S2). The *R* ^2^ values as function of the slope of the incidence curve for both males and females are shown in Figure 2d,e.

The three‐parameter model improves on the two‐parameter model by also describing the mildly age‐related disease code incidence: 94% of the codes show *R* ^2^ > 0.9 (<*R* ^2^> = 0.96, median *R* ^2^ = 0.98). The typical width of the $X_{c}$ distribution in these cases is about σ = 1–2. Examples include type‐2 diabetes, acute pancreatitis, and obstructive chronic bronchitis (Figure 2b). Many of these diseases have strong risk factors (nutrition, smoking, and so on) which may contribute to the variance of $X_{c}$ in the population. The best‐fit parameters and *R* ^2^ values for all disease codes are provided in supplementary files S1–S2.

The model does not describe well the incidence of a few common age‐related diseases. A notable example is osteoporosis in women (Figure 2c). The incidence curve rises sharply after age 50, in a way that the model cannot capture. Interestingly, osteoporosis in men is well described by the model (Figure 2c). This suggests that effects such as menopause‐related changes go beyond the current framework.

Another case in which the model does not capture the incidence curve is Alzheimer's disease and dementia. These diseases have an exceptionally large slope of about 20% per year. The model can only explain this large slope with a disease threshold $X_{c}$ that exceeds the threshold for mortality. Figure 2c shows the best fit with the maximal $X_{c}$ values equal to that of mortality ($X_{\mathrm{death}}=17$), showing an underestimate of the slope. This suggests that the age‐related factor X in these brain diseases might be distinct from total body senescent‐cell level (Bussian et al., 2018; Zhang et al., 2019). A better fit is achieved when allowing $X_{c}$ to exceed 17 (black lines in Figure 2c).

### 2.3 The model also captures disease incidence from UKBiobank

As an independent test, we considered incidence curves from a second large dataset, UKBiobank (Sudlow et al., 2015). Here, 202,333 men and 240,260 women reported the age of incidence of 445 diseases. We considered the 79 (female) and 61 (male) diseases reported by more than 1000 people. Of these, 43 (male) and 54 (female) are at least mildly age‐related and 25 (male) and 28 (female) are strongly age‐related, as defined above.

In UKBiobank data, as in the Clalit data, the two‐parameter model describes well the strongly age‐related ICD9 codes: 92% of the codes show *R* ^2^ > 0.9 (<*R* ^2^> = 0.97, median *R* ^2^ = 0.97). The three‐parameter model also describes the mildly age‐related diseases: 96% of the codes show *R* ^2^ > 0.9 (<*R* ^2^> = 0.98, median *R* ^2^ = 0.98). The model parameters, *R* ^2^ values, and the incidence curves provided in supplementary files S3–S4.

### 2.4 Incidence of idiopathic pulmonary fibrosis and osteoarthritis can be explained by threshold‐crossing of the ratio of progenitor cell removal to proliferation rates

We next focus on several classes of pathologies and provide, for each case, a specific mechanism for the threshold‐crossing assumed in the model. We begin with two well‐known age‐related diseases, idiopathic pulmonary fibrosis (IPF) and osteoarthritis (OA). Both are progressive diseases whose origin is currently debated. We will suggest a physiological parameter *ϕ* for these diseases and show how senescent‐cell level can affect this parameter.

**FIGURE 3:**
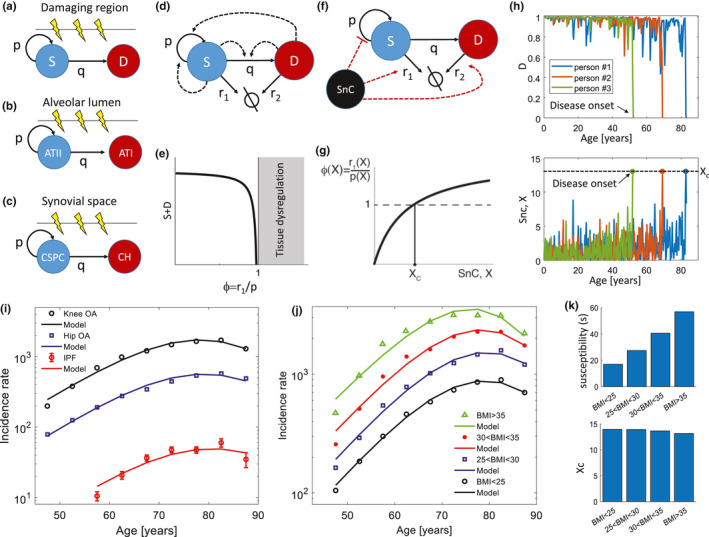
Threshold‐crossing of the ratio of progenitor removal to proliferation can explain the incidence of idiopathic pulmonary fibrosis and osteoarthritis. (a) General scheme of “frontline” tissues, in which stem or progenitor cells, S, are as exposed to damage as their differentiated progeny, D. (b) The lung alveoli progenitor AT2 cells lie within the same layer as the differentiated AT1 cells. (c) In joints, cartilage‐derived stem/progenitor cells (CSPC) are at the superficial zone and face the same amount of damage as the differentiated chondrocytes (CH). (d) Homeostasis is maintained by signals secreted from the cells that act on the proliferation and differentiation rates. (e) When the physiological parameter *ϕ* = *r*_1_/*p*, the ratio of progenitor removal and proliferation rates, exceeds *ϕ*_c_ = 1, the number of cells in the tissue, S + D, crashes. (f) Senescent cells slow progenitor proliferation due to SASP from both local and systemic senescent cells (SnC). Senescent cells can also disrupt the extracellular matrix and increase removal rate *r*_1_. (g) When senescent cells cross a threshold $X_{c}$, tissue collapse is predicted to occur. (h) Simulated tissue dynamics show that when senescent cells cross a threshold, the number of differentiated cells collapse, triggering the onset of the disease. (i) The model fits the incidence curves of IPF (Navaratnam et al., 2011) and OA (knee and hip) well. (j) Incidence of knee OA stratified by BMI, see Figure S4 for hip OA. (k) Effect of BMI on best‐fit parameters for knee OA incidence, with s in percent. OA data from (Reyes et al., 2016)

Both diseases occur in tissues, which, for structural reasons, are constrained to have progenitor cells that are exposed to damage (Figure 3a). We call this situation a *“frontline” tissue*. IPF occurs in the lung alveoli, which are one cell‐layer thick, to allow for efficient gas exchange. The progenitor cells, called AT2 cells, lie within the same layer as the differentiated cells, called AT1 cells (Desai et al., 2014; Logan & Desai, 2015; Nabhan et al., 2018) (Figure 3b). Thus, progenitors are as exposed to damage as their differentiated progeny.

Another frontline tissue relates to osteoarthritis (OA), which occurs in chondrocytes in joints such as knees and hips. Here, progenitors are at the synovial surface of the joint (superficial zone) (Dowthwaite et al., 2004; Jiang & Tuan, 2015) and face the same amount of mechanical damage as the differentiated chondrocytes (Figure 3c).

Frontline tissues can be contrasted with tissues in which stem cells are protected from damage, such as bone marrow in which hematopoietic stem cells are protected inside bones, skin in which stem cells lie below several layers of epithelial cells, or the intestine, where stem cells reside at the bottom of the crypt, protected from luminal contents.

In IPF, lung alveolar cell populations decline, and lung function drops to lethal levels within few years of onset (Raghu et al., 2002). Similarly, in OA, cartilage is progressively lost over many years in certain regions of the joint (Vincent et al., 2012). In this section, we provide a general mechanism that can intrinsically cause a collapse of such frontline tissues with age and explain the incidence curves of these diseases.

In frontline tissues, as in other tissues, the progenitor cells must proliferate to renew their own numbers as well as to provide differentiated cells (Figure 3d). They must maintain homeostasis, namely, proper amounts of progenitor and differentiated cells. Homeostasis is maintained by feedback signals secreted from the cells that act on the proliferation and differentiation rates (Figure 3d). For example, differentiated cells often signal with TGF‐β to affect the differentiation rate (Chen et al., 2018; Zhao et al., 2013).

In frontline tissues, homeostasis is harder to achieve than in tissues in which progenitor cells are protected, because of the higher rate of removal of progenitor cells. No matter what the feedback circuits for homeostasis are, a catastrophe happens when progenitor removal rate *r*_1_ exceeds the maximal progenitor proliferation rate *p* (for proof see SI section 5). In this case, there are not enough progenitor cell divisions to populate the tissue and the tissue collapses (Figure 3e). The rate of this collapse depends on the removal rates of the cells, and thus can be different in different tissues. After the collapse, tissue repair cannot proceed by regeneration and instead must rely on processes such as fibrosis, migration, and metaplasia, but this repair reduces tissue function and pathology occurs.

The relevant physiological parameter is thus *ϕ* = *r*_1_/*p*, the ratio of removal and proliferation rate of the progenitor cells. Disease onset occurs when *ϕ* exceeds *ϕ*_c_ = 1 (Figure 3e). This is criterion (i) of the model.

Senescent cells affect proliferation and removal in a way that tends to increase *ϕ* (Figure 3f). Senescent cells slow down progenitor proliferation due to the factors in the SASP (Coppé et al., 2010) from both local and systemic senescent cells. Senescent cells can, in some tissues, also disrupt the extracellular matrix and increase removal rate *r*_1_ (Jeon et al., 2018). Thus, when senescent cells cross a threshold $X_{c}$, tissue collapse is predicted to occur in the susceptible population (Figure 3g). Such a collapse occurs in simulations of tissue homeostasis circuits coupled with stochastic senescent cell dynamics (Figure 3h). This is criterion (ii) for the model, providing a basis for disease onset when $X>X_{c}$.

Indeed, the incidence of IPF (Navaratnam et al., 2011) and OA (Reyes et al., 2016) is well described by the two‐parameter model (Figure 3i, Figure 2a, *R* ^2^ > 0.95). The model also describes well incidence curves for OA in different joints (Figure S3, <*R* ^2^> = 0.96, *R* ^2^ > 0.93).

The present frontline scenario may explain how IPF and osteoarthritis begin. It makes two main predictions: That diseases should start in the part of the tissue with largest removal rate, and that environmental and genetic risk factors should increase the removal rate of progenitors.

The first prediction is met by both IPF and OA. Both diseases occur in the part of the tissue with largest mechanical stress, and hence highest removal rate *r*_1_. OA occurs in the part of the joint that bears the most weight (Vincent et al., 2012), and IPF begins at the outside of the lung (Raghu et al., 2011) which has highest alveolar expansion. This agrees with the theory, because at these locations *ϕ* is highest and most likely to exceed *ϕ*_c_.

The second prediction is also met in OA and IPF: The susceptible population includes those bearing genetic or environmental factors that increase removal rate of progenitor cells in the specific tissue. In IPF, genetic factors include genes needed for AT2 function such as surfactant and telomerase (Kropski et al., 2015), and mucin genes that, when mutated, impair particle removal by the bronchi and increase damage to alveolar cells (Yang et al., 2015). These factors increase *r*_1_, and thus increase *ϕ*. Environmental factors include smoking and damaging agents, which increase cell removal in the alveoli. In OA, high BMI and asymmetry in weight distribution of the joints are risk factors. These factors increase the stress on the joints and increase *r*_1_, increasing *ϕ*.

Indeed, the present model can describe incidence curves of subpopulations with different risk factors. We analyzed the incidence of OA in knee and hip in populations with different BMI from (Reyes et al., 2016). We find that the incidence curves are well described by the two‐parameter model and that the main effect of BMI is on the susceptibility parameter s, which varies about 3‐fold between BMI below 25 and above 35 (Figure 3k, for hip OA see Figure S4). The threshold $X_{c}$ did not vary appreciably with BMI. Similar results are obtained from Clalit data (Figure S5).

Notably, this picture is independent of the precise feedback loops that maintain homeostasis (SI section 7). To demonstrate this, we simulated a wide range of feedback mechanisms that can provide homeostasis to a tissue with a progenitor cell S and a differentiated cell D (Kunche et al., 2016; Lander et al., 2009; Yang et al., 2015b, 2017). We scanned all possible combinations of feedback loops (the dashed arrows in Figure 3d, each can be positive, negative, or zero, leading to 81 possible mechanisms) and found a class of 17 homeostatic mechanisms that provide stable cell populations (Figure S7). We next simulated senescent‐cell stochastic trajectories, and modeled the effects of senescent cells as a reduction in S proliferation rate (SI section 7, Figure S8). The incidence of tissue crash events, in which D cells populations collapse (Figure 3h, see also Figure S8,9), is well described by the two‐parameter model, in excellent agreement with the observed incidence.

### 2.5 Cancer incidence can be explained by threshold‐crossing of the ratio of cancer growth rate to removal rate

We next consider the case of cancer and analyze what physiological parameter *ϕ* might provide the incidence curves for different cancers. Cancer cells arise continuously in the body due to accumulation of mutations (Omenn, 2016). These mutant cells are removed by immune surveillance, primarily by NK cells and macrophages, and at later stages by T cells. If the cancer cells manage to grow to a critical amount of roughly 10^6^ cells, they organize a local microenvironment that downregulates further immune clearance (McBride & Howie, 1986; Palmer et al., 2018).

**FIGURE 4:**
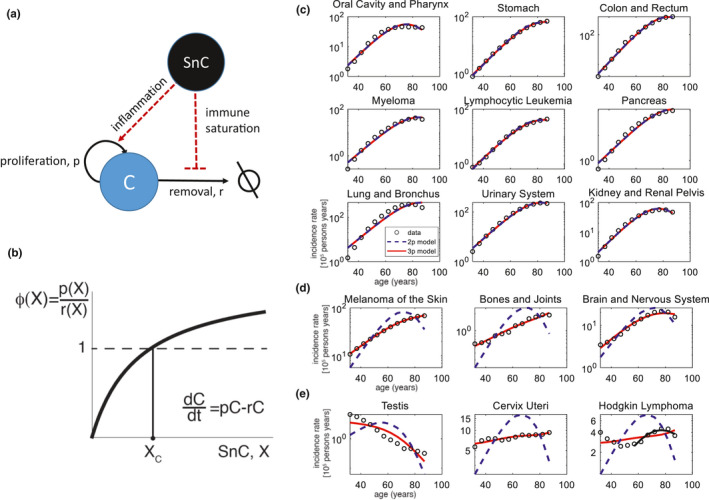
Cancer incidence can be explained by threshold‐crossing of the ratio of cancer growth rate to removal rate. (a) Cancer cells C proliferate at rate *p*, and are removed at rate *r*. With age, rising senescent‐cell (SnC) levels cause immune saturation by taking up some of the removal capacity of NK cells and macrophages (Karin et al., 2019). Inflammation driven by senescent cells increases proliferation p for some cancer types. (b) Both effects, raising p and lowering removal r, cause the parameter *ϕ* to increase, *ϕ*(*X*) = *p*(*X*)/*r*(*X*). Thus, there exists a threshold $X_{c}$ where *ϕ* exceeds the critical value of 1 and cancer cells proliferate more than they are removed, reaching a clinically detectable disease. (c) The models fit various types of cancer very well. (d) Example of cancer types in which the three‐parameter model provides an excellent fit but the two‐parameter model does not. (e) Example of cancer types not described by the models. In the case of Hodgkin Lymphoma, the model describes well the incidence curve above age 50 (black line)

Consider cancer cells that proliferate at rate *p* and are removed at rate *r* (Figure 4a). The rate of change of the number of cancer cells *C* is as follows:

$$\frac{\mathrm{dC}}{\mathrm{dt}}=pC‐rC$$

Cancer grows if proliferation exceeds removal, *p* > *r*. We can thus define the relevant physiological parameter as the ratio between growth and removal rates: *ϕ* = *p*/*r*. The critical threshold for cancer onset thus occurs at *ϕ*_c_ = 1. At this threshold, growth equals removal.

The parameter *ϕ* is affected by senescent cells, which affect both p and r (Figure 4a). Interestingly, the main effects are opposite to the case of frontline tissues discussed above. With age, the rising senescent‐cell level takes up some of the immune removal capacity of cancer. For example, NK cells remove senescent cells, and thus are occupied with or exhausted by senescent cells and can presumably do less of their cancer‐removing roles. Note that NK cell numbers do not significantly change with age in humans (Alpert et al., 2019; Valiathan et al., 2016). Thus, removal rate *r* drops with senescent‐cell level *X*, *r* = *r*(*X*). We term this proposed effect “immune saturation,” where there are so many senescent cells that they occupy the parts of the immune system that remove them, and thus overwhelm the capacity of the same immune cells to remove sporadic cancer cells.

Other effects of senescent cells, such as chronic inflammation, raise mutation rates and proliferation rate p for some cancer types (Bavik et al., 2006; Coussens & Werb, 2002; Davalos et al., 2010; Krtolica et al., 2001; Liu & Hornsby, 2007). Both effects, raising p and lowering removal *r*, cause the parameter *ϕ* to increase with senescent cell load, *ϕ*(*X*) = *p*(*x*)/*r*(*x*). Thus, there exists a threshold $X_{c}$ where *ϕ* exceeds the critical value of 1 and cancer cell proliferation exceeds removal, reaching a clinically detectable pathology (Figure 4b). Thus, we have criteria (i) and (ii) for the model, with cancer onset when $X>X_{c}$.

Individuals susceptible to a given type of cancer have a low threshold $X_{c}$. This low threshold can arise from genetic factors (e.g., BRCA mutations for breast and ovarian cancer) and environmental factors (such as smoking for lung cancer and UV exposure for skin cancer) that generate more occurrences of the cancer cells in the tissue. The low threshold can also be due to bad luck, a rare mutation or combination of mutations that arises by chance. Each pre‐cancerous site has a different proliferation rate *p* and removal rate *r* depending on the local niche and the mutational and epigenetic background of the cell. Hence, the more occurrences of cancer in the tissue, the higher the maximal *ϕ* among all occurrences. This lowers the threshold of senescent cell level needed for cancer onset.

We compared the model to data on the incidence of 100 cancer types from the SiteSEER database (National Cancer Institute et al., 2018). Of these cancers, 87 are at least mildly age‐related as defined above. Of these, we find that 66 are well described by the two‐parameter model (*R* ^2^ > 0.9) (Figure 4c). This agreement is similar to that of the previously proposed IMII model for cancer, with the added benefit that the present model captures the decline at very old ages. The typical values of $X_{c}$ are 13–15, and the susceptibilities, s, range from 10^−4^ to 0.1. All cancer incidence curves from the SiteSEER database are shown in Figure S10. The best‐fit parameters and *R* ^2^ values are provided in supplementary file S5.

The three‐parameter model improves significantly on the two‐parameter model in 15 types of cancer and describes well 81 cancer types (*R* ^2^ > 0.9). In these 15 cancers, the slope of incidence with age is relatively low (mean 3%, only mildly age‐related). The width of the $X_{c}$ distribution is about σ = 3 for these cancers. Examples of incidence curves are shown in Figure 4d. Interestingly, skin cancers including melanoma are among the cancers predicted to have a broad distibution of $X_{c}$. One explanation is the relatively wide range of UV exposure in the US population included in the database due to a variety of climates, which potentially creates different thresholds in different individuals.

Other cancers described better by the three‐parameter model include those with sizable incidence at young ages. This includes cancers of bone and nervous system. This young‐onset contribution effectively decreases the slope of incidence with age, which is captured by the model as a wide range of $X_{c}$.

There are several types of cancer that are not fit well by either the two‐ or three‐parameter models (12 cancer types with *R* ^2^ < 0.9, 5 cancer types with *R* ^2^ < 0.8, Figure 4e). These include cancers which are most common at young ages, such as testicular cancer whose occurrence drops with age, and cervical cancer, which has a viral origin. The rest of the cancers that are poorly fit have a bimodal age distribution, with a peak at young ages and then an age‐related rise above middle age. These include lymphomas such as Hodgkin's lymphoma. The model in this case does not capture the early peak but describes incidence well if the fit is done only at ages above 50 (*R* ^2^ > 0.9, Figure 4e black line).

### 2.6 Disease incidence can be reduced by infrequent and partial removal of senescent cells starting at old age

The present model provides an opportunity to predict the effects of treatments that remove senescent cells. Such treatments have been demonstrated in mice, including senolytic drugs that kill senescent cells (Hickson et al., 2019; Jeon et al., 2017; Justice et al., 2019; Palmer et al., 2019; Pignolo et al., 2020; Schafer et al., 2017b; Short et al., 2019; Xu et al., 2018), and immune therapy that causes T cells to target senescent cells (Amor et al., 2020). Since these treatments are likely to have side effects, it is desirable to give them infrequently. The present model has the advantage of accounting for the senescent‐cell re‐accumulation process, and thus, the frequency of the treatment can be optimized to minimize re‐accumulation and maximize the interval between treatments.

We simulated the effects of removing senescent cells on the incidence of a representative age‐related disease ($X_{c}=14,s=0.1$). Similar conclusions are found for all age‐related diseases. We used a conservative approach, by assuming that only 25% of the senescent cells are vulnerable to the treatment (Karin et al., 2019). Another way of viewing this is that the treatment can remove only 25% of the damaged cells associated with the disease incidence.

**FIGURE 5:**
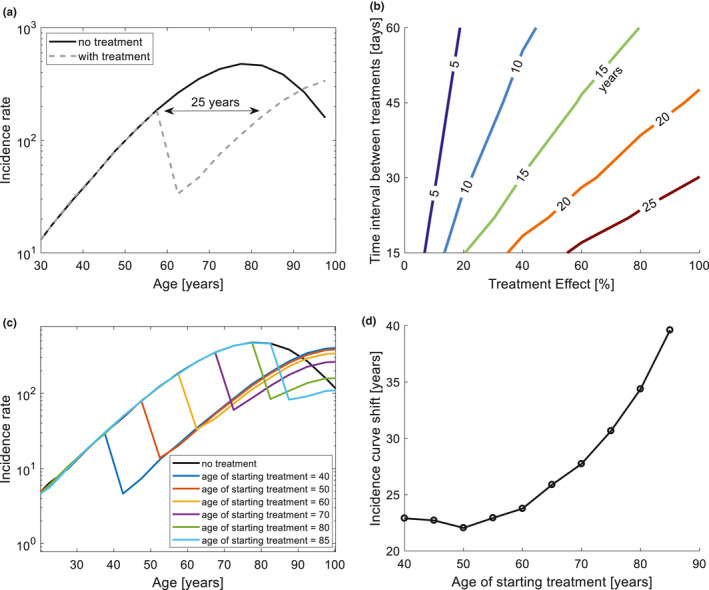
Infrequent treatment that removes senescent cells starting at old age can reduce disease incidence in the model. (a) Treatment with senolytics shifts the incidence rate in the model by 25 years. We assumed a conservative case in which only 25% of the senescent cells are drug‐sensitive (Karin et al., 2019). In this example, the treatment is given every 30 days, and starts at the age of 60 years. We used typical disease parameters ($X_{c}=14,\;s=0.1)$ to calculate the incidence curves. (b) Shift of the incidence curve to younger ages (years) as function of the time interval between treatments and the effectiveness of the treatment defined as the percentage of drug‐sensitive senescent cells that it removes. (c) The incidence curves for different choices of the age in which the senolytic treatment starts. (d) The shift of the incidence curve is larger the later the treatment starts. Panels c and d use the same treatment and disease parameters as panel a.

Treatment beginning at age 60, and given every 30 days, reduces disease incidence by about tenfold within a year (Figure 5a). The incidence curve is shifted to lower values corresponding to an age that is about 25 years younger (Figure 5a). Prevalence of the disease until age 90 is reduced by about 80%.

We used the model to scan the effectiveness of treatment regimens with different time intervals between treatments, ranging from 2 weeks to 2 months. We also scanned the treatment effect, defined as the percent of senescent cells killed with each treatment (out of the drug‐sensitive cells). Figure 5b shows the number of years by which the incidence curve is shifted to younger ages. Treatment as infrequent as once per 2 months, that kills only 40% of the drug‐sensitive senescent cells, shifts the incidence curve back by 10 years (light blue contour in Figure 5b). We also studied how the age at which treatment begins affects the incidence curves in the model. At all ages, the treatment shifts incidence to values found at younger ages (Figure 5c), with a larger shift the later the treatment starts (Figure 5d). We conclude that the model predicts that treatment starting at old age can prolong the healthspan by a decade or more.

## 3 DISCUSSION

We presented a mechanism to explain a nearly universal property of age‐related diseases: exponentially rising incidence that decreases at very old ages. The mechanism assumes that each pathology occurs when a physiological parameter exceeds a threshold. The senescent cells increase this parameter, leading to disease onset when the senescent cell level cross a disease threshold. Susceptible individuals have low disease thresholds. A stochastic model for senescent cell accumulation provides the observed incidence curves, calculated as a first‐passage time distribution of senescent cells across the disease threshold. We provide a specific interpretation of the physiological parameter affected by senescent cells for the cases of cancer, IPF and osteoarthritis. The model provides excellent fits to a new database of disease incidence with 50 million life‐years, as well as to UKBiobank. It predicts that removal of senescent cells starting in old age can significantly reduce disease incidence.

For idiopathic pulmonary fibrosis (IPF), we provide a new explanation for the origin of this disease of unknown etiology. Senescent cells slow down the proliferation of alveolar progenitor cells, and when this proliferation drops below their removal rate, the tissue collapses. This explains the sudden collapse of the alveoli at the outer parts of the lung, a location in which removal rate is largest. It also explains genetic risk factors such as germline variations that increase progenitor death in the alveoli.

With this approach, one can make connections between previously unrelated diseases. Thus, osteoarthritis, a disease of the joints, is suggested to be in the same class as IPF, a disease of the lungs. Both tissues have progenitors at the front line, where they are exposed to damage, in contrast to tissues in which progenitors are protected such as bone marrow and skin. Thus, the origin of OA is also suggested to result when progenitor proliferation rate drops below removal rate, caused by rising senescent cell level with age and their attendant systemic SASP. Risk factors for OA such as high BMI are suggested to increase removal rate and thereby increase the susceptibility to the disease.

Additional age‐related disease classes may be analyzed using the present approach. Important diseases with high prevalence including atherosclerosis and type‐2 diabetes are thought to be mediated, in part, by inflammation. SASP includes many inflammatory factors. Therefore, the theory might be extended to describe the evolution of atherosclerosis and metabolic syndrome. For example, late‐stage type 2 diabetes is associated with collapse of beta‐cell function. This collapse has been modeled as a threshold‐crossing event in which rising glucose causes glucotoxicity, making the removal of beta cells exceed their renewal (Karin & Alon, 2017; Topp et al., 2000). Since senescent cells reduce beta‐cell proliferation, and increase insulin resistance through inflammation and impact on adipocytes (Palmer et al., 2019), they can instigate this collapse and explain part of the age‐related incidence. Another class of diseases includes age‐related mortality from infectious disease (Palmer et al., 2018). Infections can be analyzed in a similar way to cancer, where the physiological parameter is the ratio of pathogen growth and removal rates. Finally, fibrotic diseases such as liver cirrhosis and focal glomerular sclerosis (a cause of end‐stage kidney disease) may also correspond to a threshold‐crossing phenomenon. The threshold‐crossing was described by a recent theoretical analysis (Adler et al., 2019) of the dynamics of myofibroblasts and macrophages. Above a threshold, the dynamics flow to a fibrosis state in which myofibroblasts and macrophages support each other at high cell concentrations (Figure S11). Senescent cells can induce such threshold‐crossing by means of pro‐inflammatory SASP (See SI section 9), increasing the range of micro‐injuries which result in fibrosis.

The current model is simplistic in assuming that a single factor, senescent cells level *X*, accounts for the incidence of all age‐related pathology. Obviously reality is more complex, and additional forms of age‐related damage and decline contribute (De Bourcy et al., 2017). There may be different populations of senescent cells at play, with differential importance of local versus systemic senescent cells in different pathologies. The lack of fit to brain degenerative diseases in this study may indicate that the brain has its own primary form(s) of damage, perhaps senescent glia, which is distinct from whole body senescent‐cell level.

We hypothesize that the present framework might apply beyond senescent cells also to other forms of age‐related damage causal for diseases. The variable *X* can be interpreted more broadly as damaged cells which incite inflammatory signals. The requirements are that, as in the SR model, damage production rises linearly with age and damage saturates its own removal. For example, DNA mutations accumulate with age and can cause cellular damage, which is removed by repair systems which have a finite capacity. A similar situation may apply to protein aggregate accumulation and for some lipid metabolic waste products (lipofuscins), which are known to accumulate with age. Thus, even if senescent cells are removed, aging and age‐related pathology will still occur albeit with a delay due to such additional factors.

The model makes several experimentally testable predictions. The first prediction is a tight relationship between senescent cells in an individual and the onset of pathology in the same individual. Future experiments in which both pathology and senescent cell level are evaluated in the same organism can shed light on the strength of the relation between senescent cells and disease onset. Another prediction concerns the *geroscience hypothesis*, which asserts that any intervention that retards the aging process will simultaneously delay the onset of multiple diseases (Barzilai et al., 2016; Franceschi et al., 2018; Kaeberlein, 2017; Kennedy et al., 2014; Kritchevsky & Justice, 2020). The model predicts that interventions that remove senescent cells, slow senescent‐cell production or attenuate SASP will have a specific and predictable global effect on the incidence of all age‐related diseases. Such treatments are predicted to affect disease incidence even when treatment is started at old age (Figure 5). Human clinical trials with senolytics are at early stages, but may in the long term allow estimation of the effect of removing senescent cells on incidence curves. Other drugs proposed to slow aging such as metformin (Barzilai et al., 2016) may attenuate senescent‐cell production (Jadhav et al., 2013) or SASP (Moiseeva et al., 2013; Saisho, 2015) and have similar effects. The wide range of mouse disease models that show improvement upon senolytic treatment tends to qualitatively support such a prediction.

## 4 METHODS

### 4.1 Incidence from Clalit database

We used Clalit health‐service electronic health records (EHR) dataset (Balicer & Afek, 2017). All incidence curves and ICD9 codes are provided in https://doi.org/10.7910/DVN/LS3WYI . For more details, see SI Section 3.

### 4.2 Analytical formula for two‐parameter model

An approximate formula for the incidence of the disease is (see SI section 2 for details):

(1) $$I\left(t\right)=sA\frac{e^{\mathrm{at}}}{1+be^{\mathrm{at}}}{\left(s+\left(1‐s\right){\left(\frac{be^{a_{d}t}+1}{b_{d}+1}\right)}^{‐\frac{A_{d}}{a_{d}b_{d}}}{\left(\frac{be^{\mathrm{at}}+1}{b+1}\right)}^{\frac{A}{\mathrm{ab}}}\right)}^{‐1}$$

with hazard function $h=A\frac{e^{\mathrm{at}}}{1+be^{\mathrm{at}}}$ and parameters a,b and *A* that depend on $X_{c}$ as follows:

$$\begin{array}{c} \text{log}A=A_{0}+A_{1}X_{c};\;\text{log}b=b_{0}+b_{1}X_{c};\\ a=a_{0}+a_{1}X_{c}\;\text{with}\;A_{0}=4.14;\;A_{1}=‐1.01;\\ b_{0}=2.24;\;b_{1}=‐0.81;\;a_{0}=‐0.0186;\;a_{1}=0.0089. \end{array}$$

The death hazard $h_{d}\left(t\right)$ has $X_{c}=X_{\mathrm{death}}=17$, giving the death parameters: $A_{d}=2.22\cdot 10^{‐6};\;b_{d}=9.774\cdot 10^{‐6};\;a_{d}=0.132.$ Since A,b,anda depend on $X_{c}$, the model depends on only two free parameters $X_{c}$ and s. Note that when s≪1, incidence is approximately proportional to s. In this limit, the age of maximal incidence rises approximately linearly with $X_{c}$ (Figure S12).

### 4.3 Analytical formula for three‐parameter model

The three‐parameter model includes a normal distribution of disease thresholds $P\left(X_{c}\right)$ with mean $\bar{X_{c}}$ and standard deviation σ. The resulting formula for the incidence is as follows:

(2) $$I\left(t\right)=\frac{\int h\left(t\right)C_{s}\left(X_{c}\right)P\left(X_{c}\right)dX_{c}}{\int C_{s}\left(X_{c}\right)P\left(X_{c}\right)dX_{c}+C}$$

where $C_{s}=s{\left(\frac{be^{\mathrm{at}}+1}{b+1}\right)}^{‐\frac{A}{\mathrm{ab}}};C=\left(1‐s\right){\left(\frac{be^{a_{d}t}+1}{b_{d}+1}\right)}^{‐\frac{A_{d}}{a_{d}b_{d}}}$. We used Equations ((1), (2)) for all results.

### 4.4 Software availability

Software that fits incidence data to the two‐ and three‐parameter models is provided in https://github.com/itaykatzir/MatlabCodeForFittingIncidence.

### 4.5 Simulation of treatment that removes senescent cells

We used the formulae developed by Karin et al. (Karin et al., 2019, supplementary note 6), and used stochastic simulations of intermittent treatment as described. We assumed that senescent cells are produced in two types: treatment sensitive and non‐sensitive. This is a conservative model, and assuming that all senescent cells are treatment‐sensitive leads to larger shifts in incidence curves to young ages.

## CONFLICT OF INTEREST

The authors declare that they have no conflict of interest.

## AUTHORS’ CONTRIBUTIONS

Itay Katzir involved in conceptualization, methodology, software, formal analysis, writing, and visualization. Miri Adler involved in conceptualization, methodology, and visualization. Omer Karin involved in conceptualization and methodology. Netta Mendelsohn‐Cohen involved in data curation. Avi Mayo involved in conceptualization, and methodology. Uri Alon involved in conceptualization, methodology, writing, and supervision.

### Open Research Badges

This article has earned an Open Data Badge for making publicly available the digitally‐shareable data necessary to reproduce the reported results. The data is available at https://doi.org/10.7910/DVN/LS3WYI.

## Supporting information

Supplementary Material

File S1

File S2

File S3

File S4

File S5

## Data Availability

The data that support the findings of this study are openly available in Harvard Dataverse at https://doi.org/10.7910/DVN/LS3WYI.
