# Supplementary material for: Senescent cells and the incidence of age‐related diseases
Source: Aging Cell. 2021 Feb 8;20(3):e13314. doi: 10.1111/acel.13314 (PMC7963340; doi:10.1111/acel.13314)
Supplement: Supplementary file 1 — Supplementary Material [file ACEL-20-e13314-s001.pdf]

Supplementary Information for **Senescent cells and the incidence of age-related diseases**

Itay Katzir[1], Miri Adler[1,2], Omer Karin[1], Netta Mendelsohn-Cohen[3], Avi Mayo[1] and Uri Alon[1]*

[1]Department of Molecular Cell Biology, Weizmann Institute of Science, Rehovot, 76100, Israel

[2]Broad Institute of Massachusetts Institute of Technology and Harvard, Cambridge, MA 02142, USA

[3]Department of Computer Science, Weizmann Institute of Science, Rehovot, 76100, Israel

*Corresponding author, uri.alon@weizmann.ac.il

Contents

Section 1. The model results hold also when senescent cells averaged over extended periods need to cross a threshold 2

Section 2. Analytical formula for two-parameter model 3

Section 3. Clalit dataset........ 4

Section 4. Model fits male incidence curves similarly to those of females. 5

Section 5. Dysregulation of front-line tissues 6

Section 6. The model describes osteoarthritis incidence in different joints in males and females. 7

Section 7. Scan of homeostatic circuits in front-line tissues 9

Section 8: Model fits well many of the 100 cancer types in the SiteSEER database 15

Section 9: Incidence of fibrosis modelled by threshold-crossing of macrophage-myofibroblast dynamics 18

Section 10: Age of maximum incidence in the model rises with disease threshold Xc. 20

Section 1. The model results hold also when senescent cells averaged over extended periods need to cross a threshold

The model in the main text marks disease onset when senescent cell levels X(t) first cross the disease threshold Xc. In this section, we consider a case where senescent cells need to exceed the threshold for an extended period for the disease to occur. As a simple model for this, we study a model variant in which senescent cells *averaged over a time period T* need to cross the threshold in order to mark disease onset.

We find that the qualitative model results hold in this case as well. For example, we calculated the average of X over T=30 days. The incidence of X crossing a threshold Xc decreases when compared to the case without averaging. This decrease is expected, because averaging decreases fluctuations in X(t) that can cross the threshold at early times. The incidence curve with averaging of X is almost identical to the incidence curve (without averaging) when the threshold Xc is recalibrated: in the example shown in Fig S1, from Xc=15 with averaging to Xc=13.3 without.

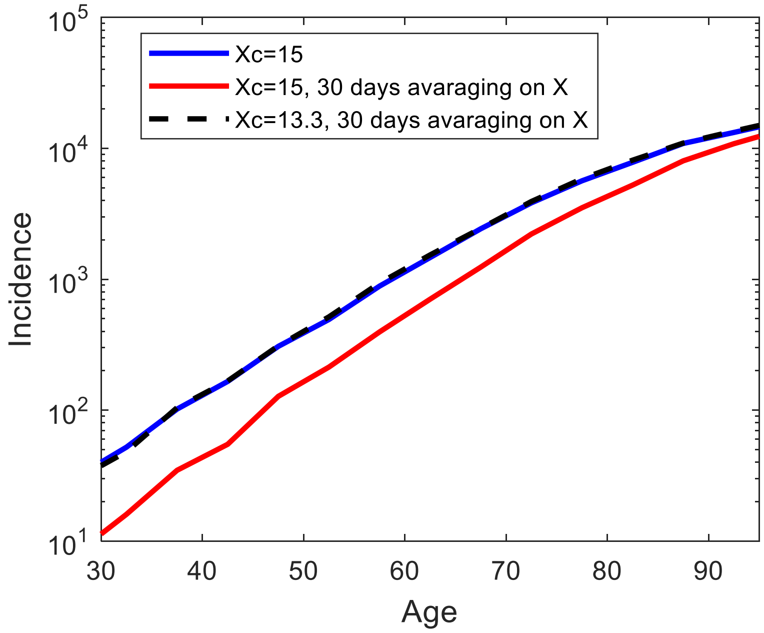

Figure S1: The effect of averaging X over time can be recalibrated by changing Xc. A model variant in which X is averaged over T=30 days shows lower disease incidence (red) than the model with the same parameters without averaging (blue). The model with averaging can be made to match the model without averaging by recalibrating the threshold Xc. Instead of Xc=15 (blue) without averaging, one can use Xc=13.3 (dashed black) which gives similar results.

Section 2. Analytical formula for two-parameter model

We derive analytical approximations for the incidence curve in the two-parameter model for a disease with threshold X_c and susceptibility s. The first passage time (FPT) hazard of X, defined as the probability per unit time that X crosses X_c at age t, denoted $h(\text{t})$, can be calculated using simulations of the SR model. We use the parameters for humans from Karin et al. [49]: $\eta = 3.683 \cdot 10^{-6}[AU]\ day^{-2}$, $\beta = 0.15\ day^{-1}$, $\kappa = 0.5[AU]$, $\epsilon = 0.142\ [AU]^2 day^{-1}$. The resulting $h(\text{t})$ can be approximated very well (R^2>0.997) by a Gamma–Gompertz function,

$$h = A\frac{e^{at}}{1 + be^{at}}$$

Whose parameters a, b and A depend on X_c as follows:
$\log A = A_0 + A_1 X_c$; $\log b = b_0 + b_1 X_c$; $a = a_0 + a_1 X_c$; with $A_0 = 4.14$; $A_1 = -1.01$; $b_0 = 2.24$; $b_1 = -0.81$; $a_0 = -0.0186$; $a_1 = 0.0089$.
The hazard for death $h_d(\text{t})$ is obtained from the SR model with the same parameters and $X_c = X_{death} = 17$, giving the death parameters: $A_d = 2.22 \cdot 10^{-6}$; $b_d = 9.774 \cdot 10^{-6}$; $a_d = 0.132$.
The incidence rate of each disease (with $X_c < X_{death}$) is defined as the number of new cases per year divided by the size of the population at risk. The susceptible population at risk is:

$C_s = s\ exp\left(-\int_0^t h(\text{t})dt\right) = s\left(\frac{be^{at}+1}{b+1}\right)^{-\frac{A}{ab}}$ where s is the susceptible fraction. This population is alive because $X_c < X_{death}$. The non-susceptible population alive at age t is:

$C = (1-s)\ exp\left(-\int_0^t h_d(\text{t})dt\right) = (1-s)\left(\frac{be^{a_d t}+1}{b_d+1}\right)^{-\frac{A_d}{a_d b_d}}$.

Thus, the incidence of the disease is:

$$(1) \qquad I(t) = h(t)\ \frac{C_s}{C_s + C} = sA\frac{e^{at}}{1 + be^{at}}\left(s + (1-s)\left(\frac{be^{a_d t}+1}{b_d+1}\right)^{-\frac{A_d}{a_d b_d}}\left(\frac{be^{at}+1}{b+1}\right)^{\frac{A}{ab}}\right)^{-1}$$

Since A, b and a depend on X_c, the model depends on only two free parameters X_c and s. We used Eq. (1) for all results of the two-parameter model. Note that when $s \ll 1$, which is the case for most of the diseases we study, incidence is approximately proportional to s:

$$I(t) \approx sA\frac{e^{at}}{1 + be^{at}}\left(\frac{be^{a_d t}+1}{b_d+1}\right)^{\frac{A_d}{a_d b_d}}\left(\frac{be^{at}+1}{b+1}\right)^{-\frac{A}{ab}}$$

In this limit, the age of maximal incidence rises approximately linearly with X_c, until X_c comes close to X_{death}, when the age of maximal incidence rises sharply (Fig S12).

Section 3. Clalit dataset

Data in the Clalit dataset was anonymized by hashing of personal identifiers and addresses and randomization of dates by sampling a random number of weeks uniformly between 0 and 13 for each patient and adding it to all dates in the patient records. Diagnosis codes were acquired from both primary care and hospitalization records, and were mapped to the ICD9 coding system. Research was conducted under Clalit Helsinki Committee 0195-17-COM2.

The ICD9 codes are arranged in a hierarchy of three levels. We considered all codes at the middle level. For example: a top-level code is *205 myeloid leukemia*, a mid-level code is *205.0 acute myeloid leukemia*, and a bottom-level code is *205.01 acute myeloid leukemia in remission*. We considered codes found in more than 10^4 persons (totaling 1274 ICD9 codes). We removed 265 irrelevant ICD9 codes (for example, codes containing "HISTORY" or "FOLLOW UP"). Incidence was computed as the ratio between number of first cases for a given ICD9 code (first for a given person) for a given sex and 5-year age bin, relative to the total number of person-years in the bin, counting only people above age 30 who are alive and active in Clalit, totaling 29 million person-years.

Errors in an individual's age have been suggested to explain some of the flattening of the human mortality curve at very old ages (Newman 2018). We therefore tested whether the disease incidence curves, and especially their decline at very old ages, might be affected by errors in the individual's age or in the time of onset of a disease. We find that even large and frequent errors cannot cause a non-declining curve to decline at old ages (data not shown).

Section 4. Model fits male incidence curves similarly to those of females.

Figure S2 is the parallel to Figure 2 in the main text, but with data for males.

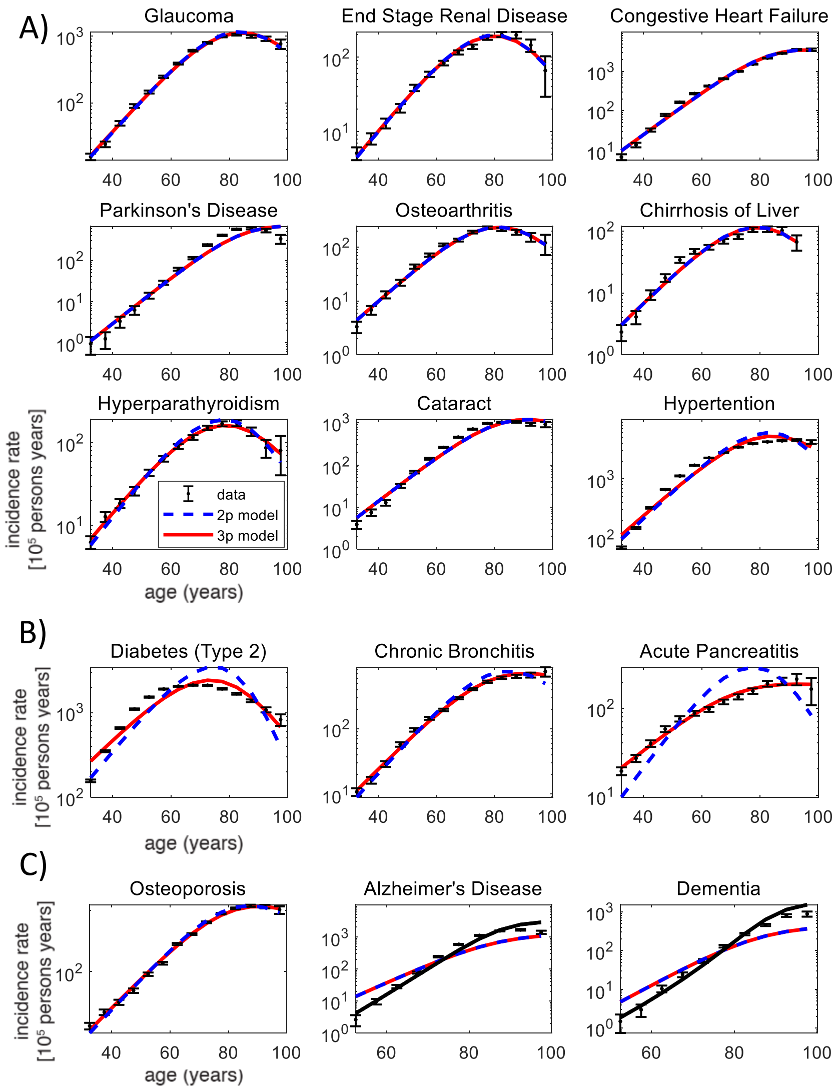

Figure S2: The model fits male incidence curves similarly to those of females. (A) The two-parameter (2p) and three-parameter (3p) models fit the incidence curves of many age-related diseases. The data presented is for males; similar results for females are shown in Figure 2. (B) For some diseases, the two-parameter model cannot describe the incidence curves, whereas the three-parameter model provide an excellent fit. (C) The model describes the incidence of osteoporosis well in men (left panel, circles), whereas osteoporosis in women is not well described (Figure 2C). The model cannot capture the incidence curve of Alzheimer disease (middle panel) and dementia (right panel), which have extremely large slopes (20% per year), using the maximal value of Xc=17 corresponding to the threshold of mortality, but the fits are improved with Xc=20 for dementia and Xc=23 for Alzheimer's disease (black lines in Figure 2C). Error bars are 95% CI assuming a binomial distribution, $CI = \pm 2\sqrt{I(10^5 - I)/P}$, where I is the incidence rate per 10^5 person-years and P is the total number of person-years in a given bin.

Section 5. Dysregulation of front-line tissues

In this section we prove that a general model of front-line tissues shows tissue collapse when stem-cell proliferation drops below stem-cell removal. We assume that stem cells S proliferate at rate p, and differentiate at rate q into differentiated cells D. The removal rate of S and D cells is $r_1=r_2=r$:

$$\frac{dS}{dt} = pS - rS - qS$$
$$\frac{dD}{dt} = qS - rD$$

Adding these two equations gives:

$$\frac{d(S+D)}{dt} = pS - r(S+D)$$

We can provide an upper bound for the right-hand side by changing S to $S + D$ because D is always positive:

$$\frac{d(S+D)}{dt} < p(S+D) - r(S+D) = (p-r)(S+D)$$

We end up with an equation for total number of cells T=S+D which goes as

$$\frac{dT}{dt} < (p-r)T.$$

When removal exceeds proliferation, $p < r$, or equivalently $\phi = \frac{r}{p} > \phi_c = 1$, the rate of change of the total cell number is negative. Total cell number $T = S + D$ goes to zero exponentially fast with time, and the tissue collapses. The rate at which the tissue collapses depends on the net rate $p - r$. Intuitively, when removal exceeds stem cell proliferation, stem cell divisions can not supply sufficient cells to the tissue to overcome removal.

Front line tissues are thus prone to collapse, since stem cells are removed at similar rates to differentiated cells. In contrast, in non-front-line tissues, whose stem cells are protected from damage, stem-cell removal rate is smaller than that of differentiated cell. Tissue crash requires a larger reduction in stem-cell proliferation than in front line tissues.

Section 6. The model describes osteoarthritis incidence in different joints in males and females.

The main text presents data for knee osteoarthritis (OA). Here we present data for hip, knee and hand. The thresholds are in the range 13.5-15.5, and prevalence is in the range 5-20%. The mean R^2 for the two-parameter model is 0.96, and R^2>0.93 for all curves.

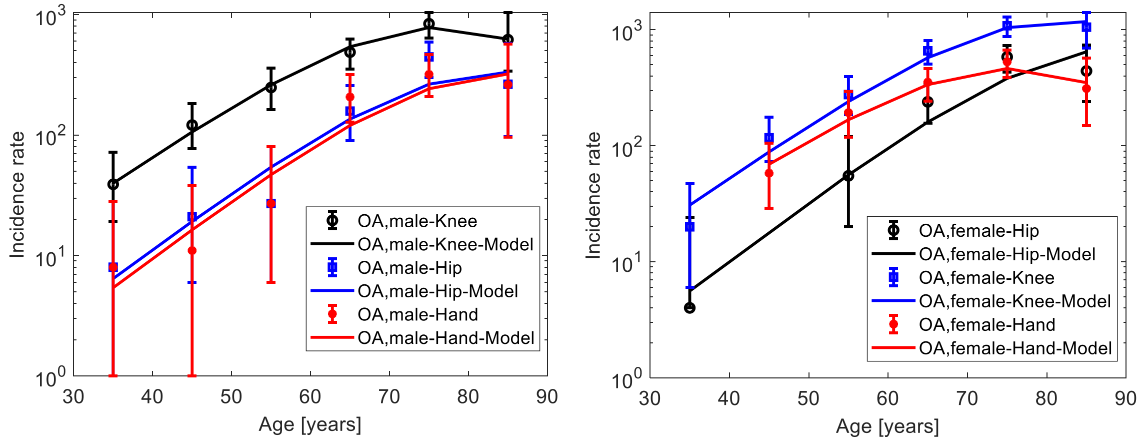

Figure S3: The two-parameter model describes well incidence curves for osteoarthritis in different joints for males and females. The observed incidence rate is from (Oliveria et al. 1995). The mean R^2 is 0.96, and R^2>0.93 for all curves.

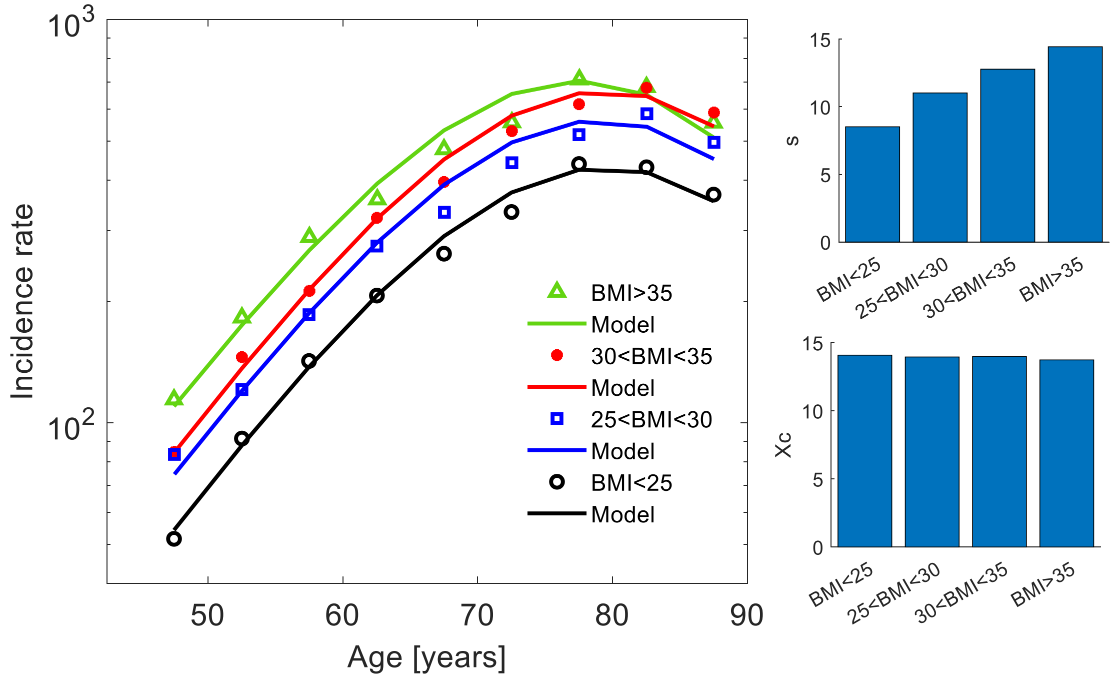

Figure S4: Incidence of hip osteoarthritis from (Reyes et al. 2016) **as a function of BMI is well described by the two parameter model.** The best fit susceptability s, the percentage of susceptible indidivuals, rises with BMI, whereas the best fit disease threshold Xc is nearly constant.

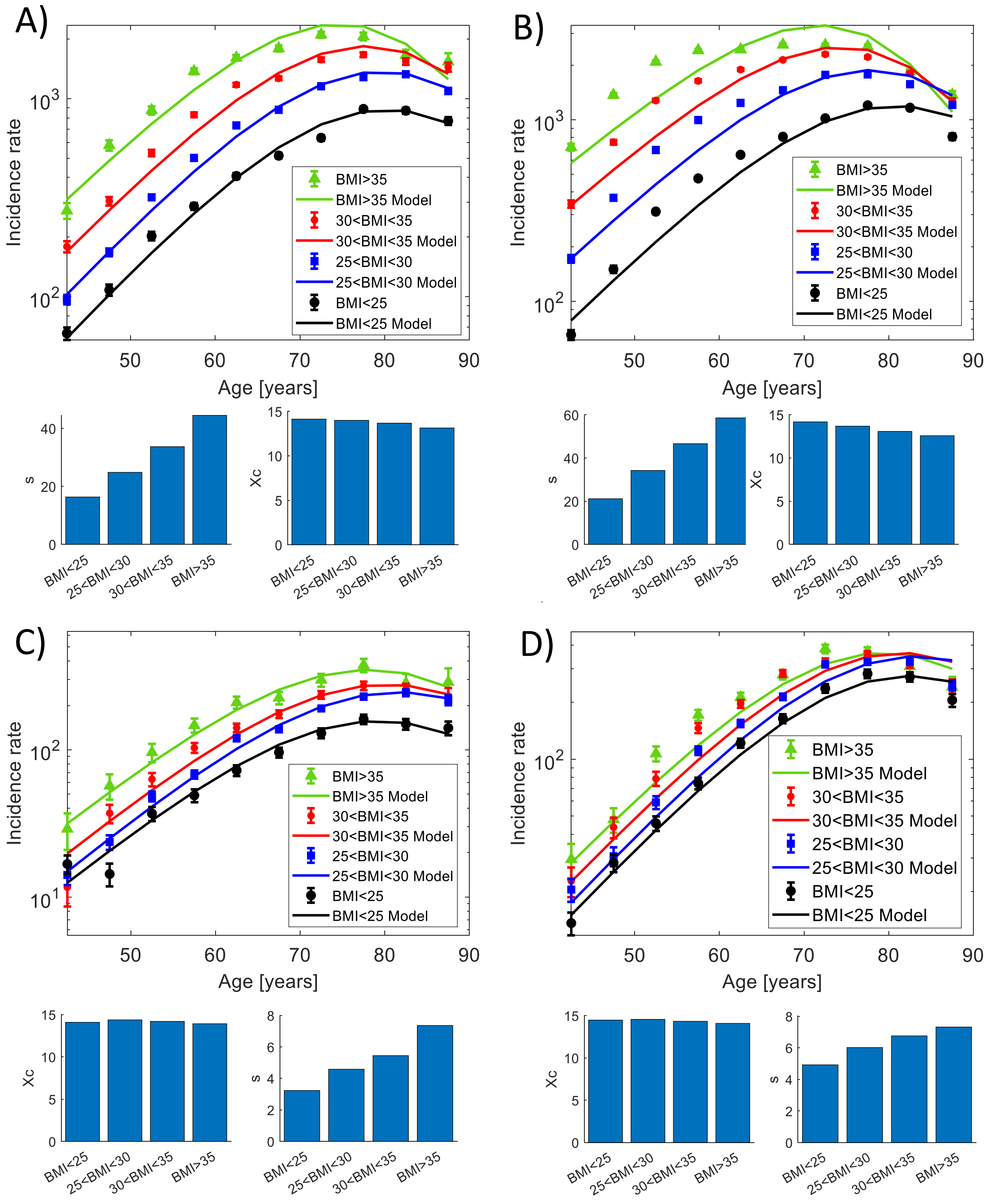

Figure S5: Incidence for knee (A,B) and hip(C,D) osteoarthritis from Clalit, as a function of BMI, for males(A,C) and females(B,D), is well described by the two-parameter model. The best fit susceptability s, the percentage of susceptible indidivuals, rises with BMI, whereas the best fit disease threshold Xc is roughly constant. Note the better fit in men than in women (<R^2 >= 0.98 vs < R^2>=0.94, <R^2=0.98 vs <R^2>0.9).

Section 7. Scan of homeostatic circuits in front-line tissues

In this section, we consider simple models of front-line tissues, in order to test the generality of the mechanism proposed in the main text for the onset of their age-related diseases. We will define a class of circuits and ask which can provide tissue homeostasis. We will then model the effect of senescent cells on these circuits and ask how tissue collapse and disease onset behaves with age.

We consider front-line tissues in which stem cells (or progenitor cells) supply a population of differentiated cells. Both progenitors and differentiated cells are removed during the normal function of the tissue. Examples include alveoli and joint chondrocytes.

We group together all dividing cells (stem/progenitor cells and transient amplifying cells) into a single cell type denoted S for stem cell. The S cells differentiate into a differentiated cell type, D, which does not divide. D and S are removed at rate r. We assume that removal rate is similar for both D and S, which is reasonable for tissues in which the stem cells are at the "front line" like the alveoli or the joint cartilage (Figure 3). In non-front-line tissues, stem cells are "protected" and are likely removed at a much lower rate than differentiated cells.

In order to maintain homeostasis (constant concentration) of D cells, the cells in the circuit must signal to each other to form feedback loops. In many tissues, for example, D cells secrete factors such as TGF-beta that enhance the differentiation rate. The S cells secrete factors such as IGF1 that affect their own proliferation and differentiation rates(Chen et al. 2018).

For the present purpose, we seek circuits that can show a homeostatic state, namely that for at least some parameter values, they have a stable steady-state with nonzero values for S and D. Mathematical models for such stable circuits for homeostasis have been studied in (Lander et al. 2009; Yang et al. 2017; Komarova 2013). Following these pioneering studies, we scan a class of circuits in which S and D communicate by secreted factors that can affect the differentiation and renewal rates (Figure 3). There are four possible interactions, each of which can be activating, inhibiting or absent, leading to 3^4=81 circuit topologies.

The dynamic equations are thus (for the case of coupled proliferation and differentiation):

$$\frac{dS}{dt} = p(S,D)\big(1 - a(S,D)\big)S - rS$$

$$\frac{dD}{dt} = p(S,D)a(S,D)S - rC$$

To arrive at these equations, we assume that secreted factors reach steady-state much faster than cell populations (due to their removal rate of minutes to hours, compared to days or more for cells). Thus, secreted factors are modelled as proportional to the cell type that secretes them. For concreteness, we model $p(S,D)$ and $a(S,D)$ using Michaelis-Menten terms and rescale S and D so that the Michaelis-Menten constant is 1. For example, if S drives its own growth (for example by secreting IGF-1 (Youssef et al. 2017)), we assume that $p(S,D) = p_0\, S/(S+1)$ and if S inhibits its own growth we assume $p(S,D) =$

$p_0/(S+1)$. We also assume the feedbacks combine multiplicatively. For example, if S drives its own growth, and D slows the proliferation rate then $p(S,D) = p_0\, S/(S+1) \cdot 1/(D+1)$.

As an example, consider the following circuit shown in Figure S6 (This is circuit 14 in Figure S7):

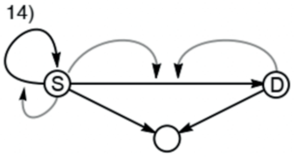

Figure S6: An example of feedback loops in a circuit that provides a stable nonzero fixed point. Interactions through secreted factors are shown in light arrows. Arrows to the open circle denote cell removal.

The equations for this circuit are:

$$\frac{dS}{dt} = p_0\, \frac{S}{S+1}\left(1 - a_0 \frac{D}{D+1}\frac{S}{S+1}\right) S - rS$$

$$\frac{dD}{dt} = a_0 p_0 \frac{S}{S+1}\frac{D}{D+1} S - rC$$

The circuit equations thus have three parameters: a_0, p_0 and r.

We find that 17 out of the 81 possible circuits can provide stable cell numbers for at least some values of a_0, p_0 and r.

To model the effect of senescent cells, we note that senescent cells secrete factors (Coppé et al. 2010), which slow down stem cell renewal (reduce stem-cell proliferation rate). We model this effect using the following Hill term:

$$p_0 \sim \frac{1}{\left(\frac{X}{X_0}\right)^n + 1}$$

Where X is the concentration of senescent cells. Here, X_0 is the concentration of senescent cells that reduces proliferation by half, and n is a steepness coefficient (we use $n = 4$). We note that a Michaelis-Menten like term (n=1) here is not sufficient to provide realistic tissue collapse curves, and thus a Hill function was used.

Senescent cells also secrete ECM-degrading factors that can in principle enhance the removal rate of D (McCulloch et al. 2017) in some tissues. We model this effect using the following term:

$$r(X) = r_0\left(1 + \frac{X}{X_0}\right)$$

We next tested whether the age-related stochastic rise in senescent cells can cause the tissues to crash. Without loss of generality, we define tissue crash when D drops to 1% of its original (homeostatic) level.

For each of the 17 circuits, we used parameters that provide a steady-state ratio of S to D equal to S/D=0.05, based on the typical value of a few percent of stem/progenitor cells in many tissues (for example, in joint cartilage (Jiang & Tuan 2015)). We used biologically plausible parameter values: removal rate *r* of 1/week-1/year, and maximal differentiation rate similar to maximal proliferation rate ($a_0 \sim 1$) as observed in experiments that add saturating TGF-beta to stem cells (Chen et al. 2018).

For each parameter set, we simulated the SR model 2000 times and used the stochastic time course of X(t) to simulate S and D dynamics. We find that the tissue crashes for all 17 circuits. The probability of crashing increases with age. We recorded the time of the crash (Figure S8A) for each of the 2000 simulated individuals. The distribution of crash times, together with a susceptible fraction *s*, provides the computed disease incidence rate. The incidence curves agree with the observed incidence curves for OA for all 17 circuits (Figure S8B,C). To check the generality of our results we also modeled the case in which the senescent cells only reduce stem cell proliferation rate, without changing the removal rate. We obtained similar results (Figure S9). We also simulated circuits in which the proliferation and differentiation are not coupled, so feedback can affect each of them separately and obtained similar results (not shown).

We note that the duration of the progressive phase of the diseases, namely the duration of time when D drops below its homeostatic value and until it drops below a pathological threshold, depends on the removal rate r. Thus, progressive phase durations of months-years (IPF) or years-decades (OA) can in principle be described using different removal rates.

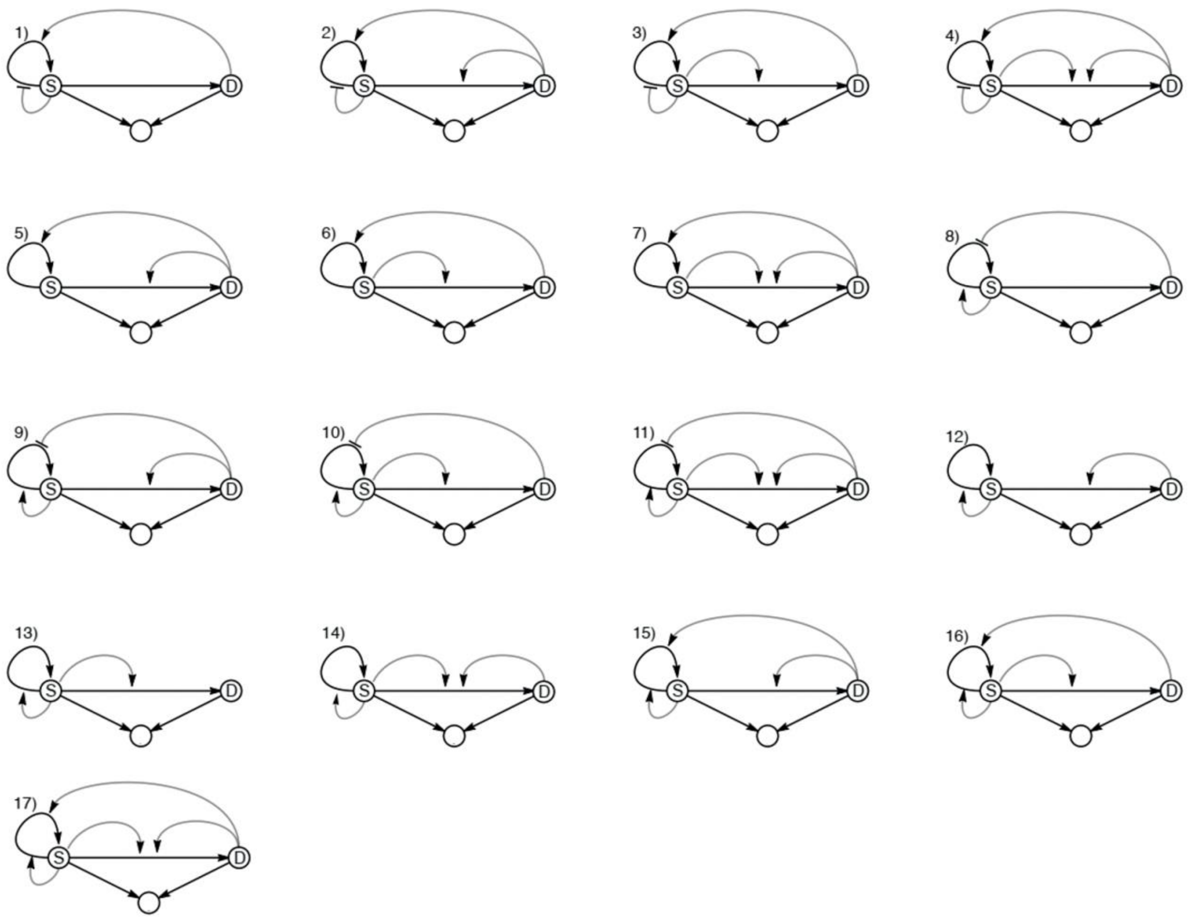

Figure S7: The 17 circuits that provide a stable nonzero fixed point for stem cells S and differentiated cells D. Arrows to the open circle denote cell removal.

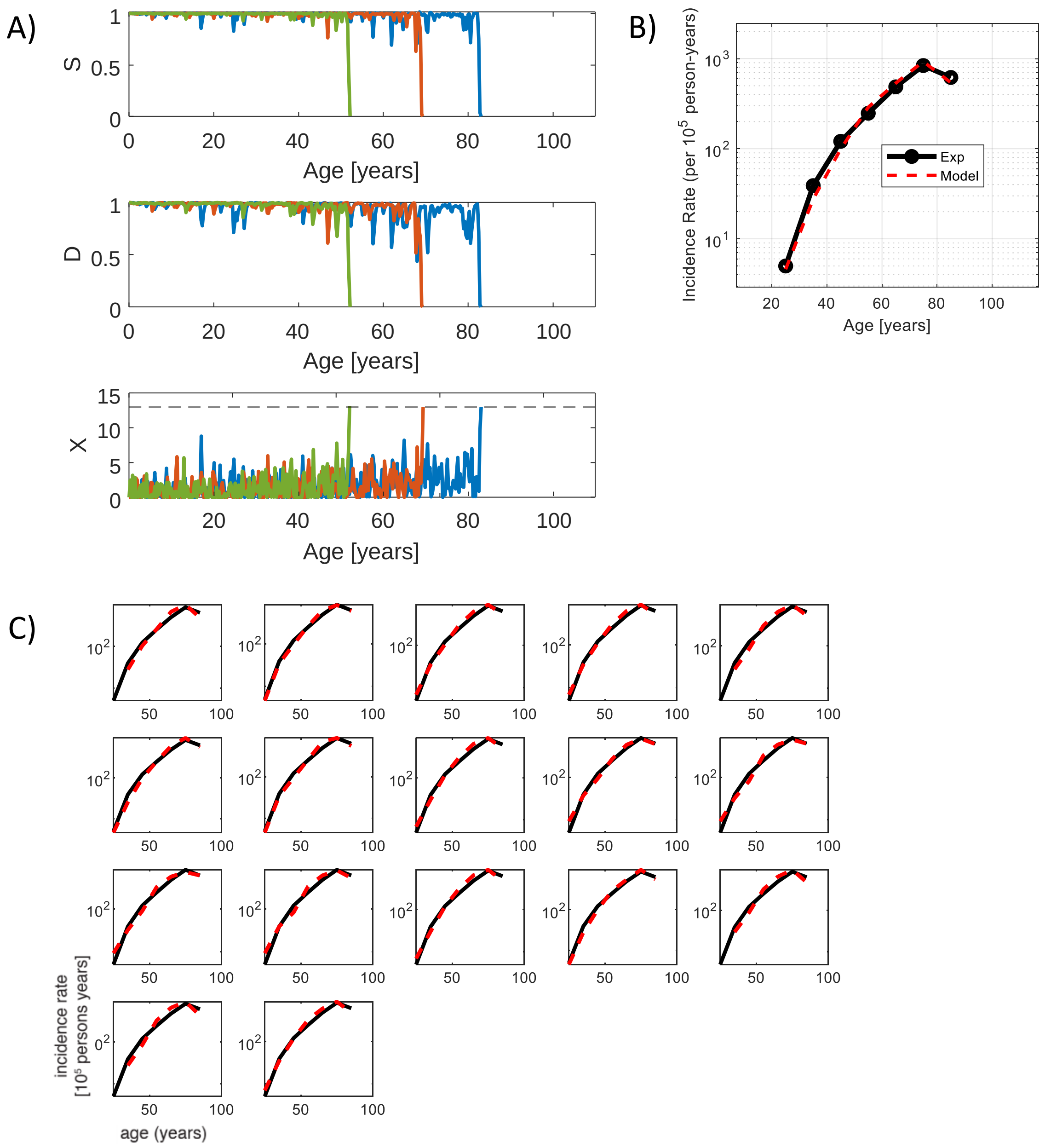

Figure S8: Tissue crash and incidence from dynamic simulations of the 17 cell circuits coupled with senescent cells. A) Trajectories of stem cells S, differentiated cells D and senescent cells X in three different simulated individuals for the case of the circuit shown in Figure S6 (which is identical to circuit 14 in Figure S7). B) Incidence curves match observed OA curve (Oliveria et al. 1995) (men, knee), with best fit model parameters of senescent-cell effect halfway concentration $X_0 = 9.75$ and the fraction of susceptible population s=0.23. The circuit parameters are $p_0 = 0.01\ [day^{-1}], a_0 = 1.08$ and $r = 7.8 \cdot 10^{-4} [day^{-1}]$. We find incidence curves very similar (R^2>0.95) to the two-parameter model and to the observed incidence curves. C) Same as B for all 17 models, each model with its best fit parameters.

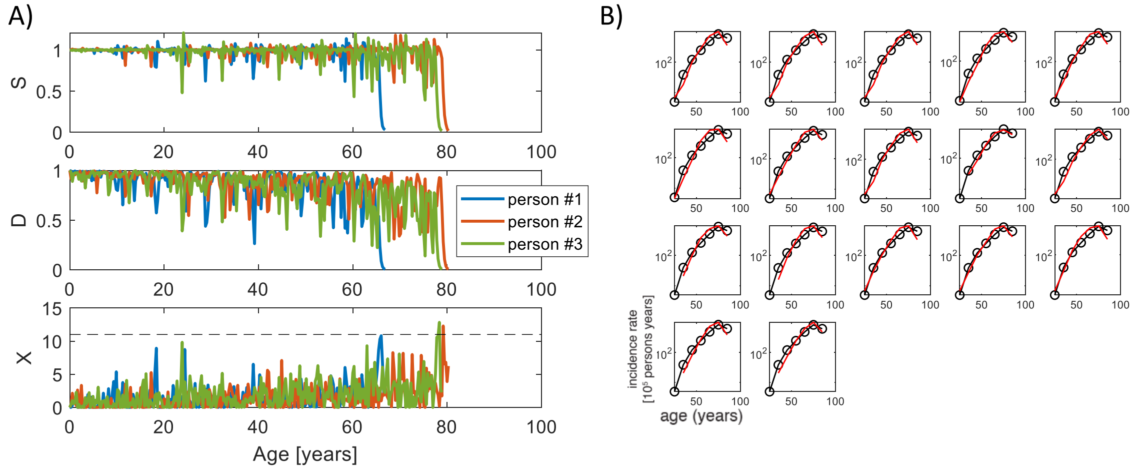

Figure S9: Dynamic simulations of the 17 cell circuits as in Figure S8 **except that senescent cells only reduce stem-cell proliferation rate, without changing the removal rate.** A) Trajectories of stem cells S, differentiated cells D and senescent cells X in three different simulated people for the case of the circuit shown in Figure S6 (which is identical to circuit 14 in Figure S7). B) Incidence curves match observed OA curve (Oliveria et al. 1995) (men, knee) for all 17 models.

Section 8: Model fits well many of the 100 cancer types in the SiteSEER database

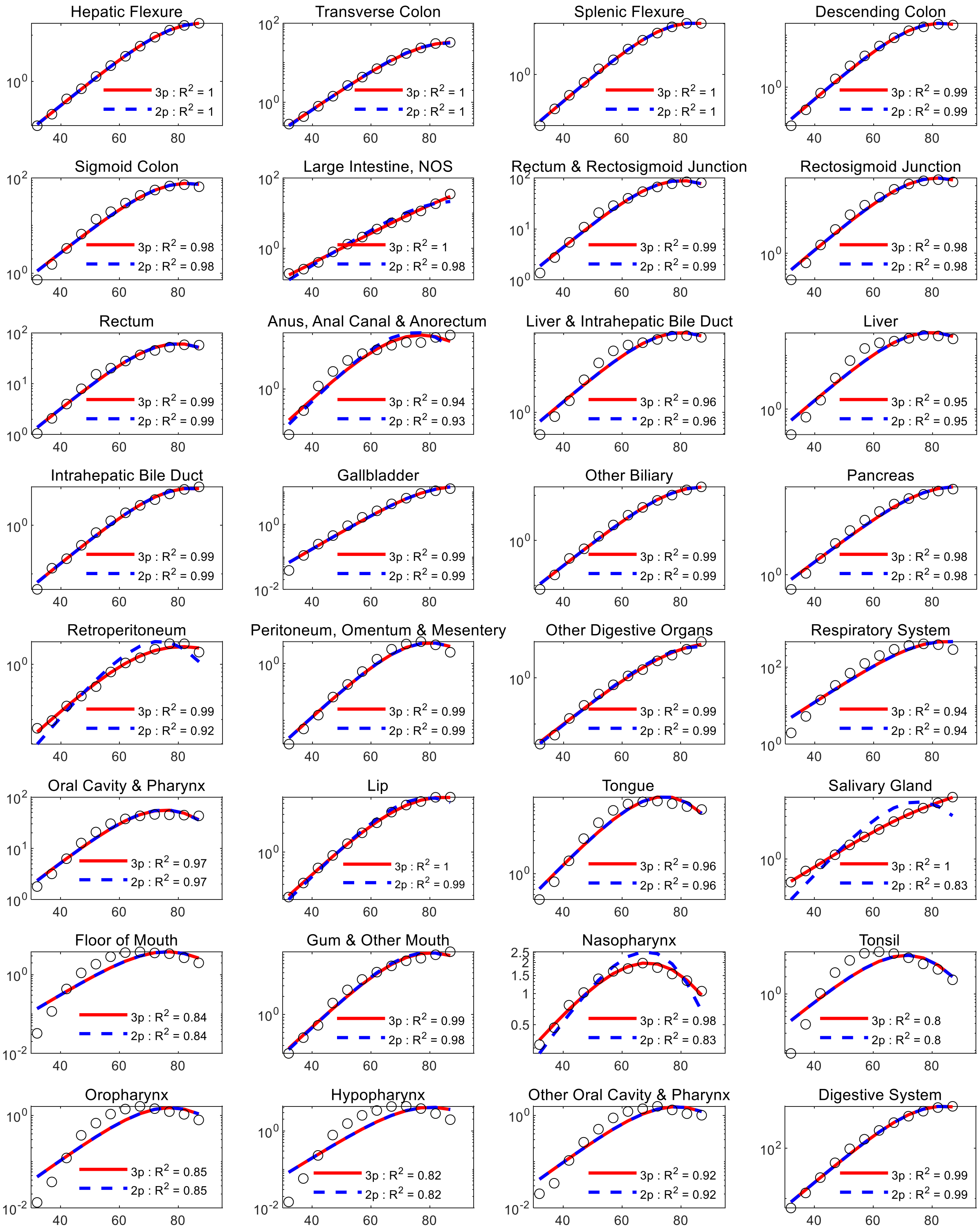

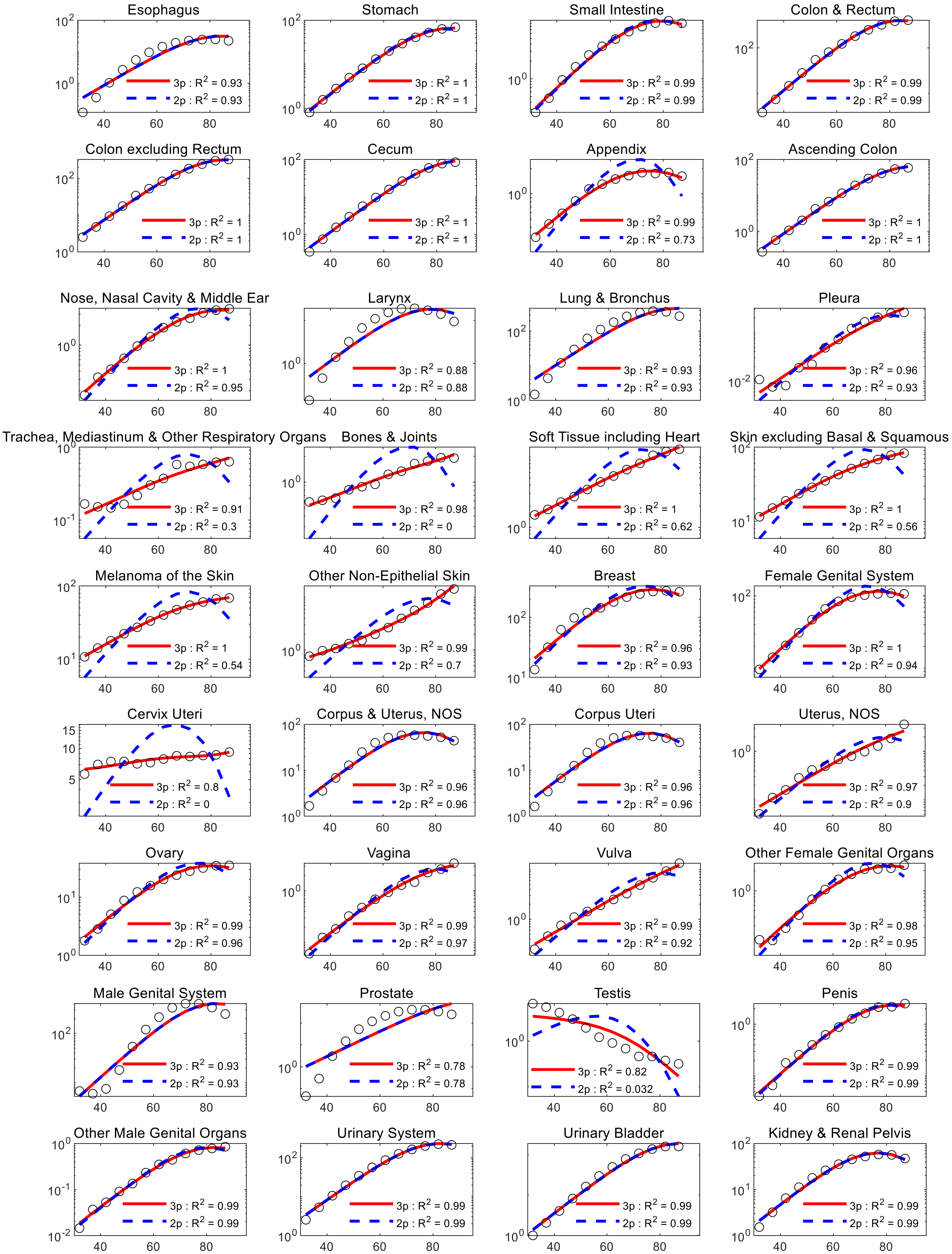
Esophagus
3p : R2 = 0.93
2p : R2 = 0.93
Stomach
3p : R2 = 1
2p : R2 = 1
Small Intestine
3p : R2 = 0.99
2p : R2 = 0.99
Colon & Rectum
3p : R2 = 0.99
2p : R2 = 0.99
Colon excluding Rectum
3p : R2 = 1
2p : R2 = 1
Cecum
3p : R2 = 1
2p : R2 = 1
Appendix
3p : R2 = 0.99
2p : R2 = 0.73
Ascending Colon
3p : R2 = 1
2p : R2 = 1
Nose, Nasal Cavity & Middle Ear
3p : R2 = 1
2p : R2 = 0.95
Larynx
3p : R2 = 0.88
2p : R2 = 0.88
Lung & Bronchus
3p : R2 = 0.93
2p : R2 = 0.93
Pleura
3p : R2 = 0.96
2p : R2 = 0.93
Trachea, Mediastinum & Other Respiratory Organs
3p : R2 = 0.91
2p : R2 = 0.3
Bones & Joints
3p : R2 = 0.98
2p : R2 = 0
Soft Tissue including Heart
3p : R2 = 1
2p : R2 = 0.62
Skin excluding Basal & Squamous
3p : R2 = 1
2p : R2 = 0.56
Melanoma of the Skin
3p : R2 = 1
2p : R2 = 0.54
Other Non-Epithelial Skin
3p : R2 = 0.99
2p : R2 = 0.7
Breast
3p : R2 = 0.96
2p : R2 = 0.93
Female Genital System
3p : R2 = 1
2p : R2 = 0.94
Cervix Uteri
3p : R2 = 0.8
2p : R2 = 0
Corpus & Uterus, NOS
3p : R2 = 0.96
2p : R2 = 0.96
Corpus Uteri
3p : R2 = 0.96
2p : R2 = 0.96
Uterus, NOS
3p : R2 = 0.97
2p : R2 = 0.9
Ovary
3p : R2 = 0.99
2p : R2 = 0.96
Vagina
3p : R2 = 0.99
2p : R2 = 0.97
Vulva
3p : R2 = 0.99
2p : R2 = 0.92
Other Female Genital Organs
3p : R2 = 0.98
2p : R2 = 0.95
Male Genital System
3p : R2 = 0.93
2p : R2 = 0.93
Prostate
3p : R2 = 0.78
2p : R2 = 0.78
Testis
3p : R2 = 0.82
2p : R2 = 0.032
Penis
3p : R2 = 0.99
2p : R2 = 0.99
Other Male Genital Organs
3p : R2 = 0.99
2p : R2 = 0.99
Urinary System
3p : R2 = 0.99
2p : R2 = 0.99
Urinary Bladder
3p : R2 = 0.99
2p : R2 = 0.99
Kidney & Renal Pelvis
3p : R2 = 0.99
2p : R2 = 0.99

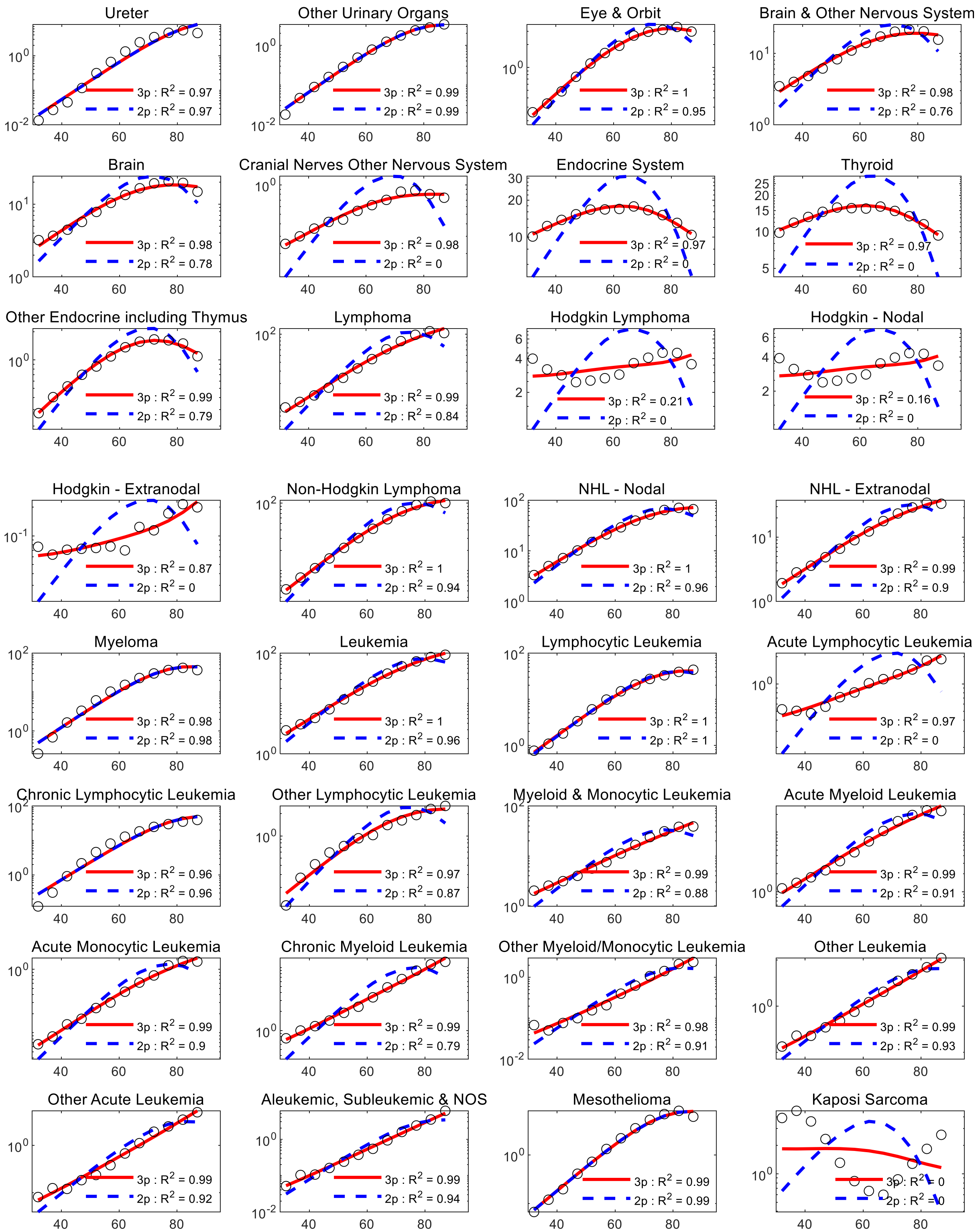

Figure S10: The incidence curves for all 100 types of cancer in SiteSEER database compared with the best-fit two- and three- parameter models. The y-axis is incidence rate per 10^5 person-years.

Section 9: Incidence of fibrosis modelled by threshold-crossing of macrophage-myofibroblast dynamics

In this section, we consider diseases that involve fibrosis: pathological, age-dependent replacement of tissue with scar fibers. We build on a recent analysis of fibrosis (Adler et al. 2019) as a circuit of interactions between the three main cell types: the damaged tissue cells D, the myofibroblasts F that lay down the scar, and monocyte-derived macrophages M. The damaged cells D activate fibroblasts into myofibroblasts, and also secrete signals that cause M cells to enter the tissue. M and F cells stimulate each other's growth by secreting growth factors (e.g. PDGF and CSF1) (Zhou et al. 2018; Adler et al. 2018).

The mutual growth-factor circuit leads to a bistable situation. A transient injury causes a brief pulse of inflammation with a brief influx of M cells (Figure S11, dashed lines). The number of M and F cells decay to zero, and normal wound healing occurs. Prolonged or repetitive injury causes the dynamics to cross a threshold, defined by a separatrix curve in the phase plane. Above this threshold, the dynamics of M and F cells flow to a state in which M and F support each other at high concentrations (Figure S11, full lines). This state is the model's equivalent of fibrosis. In some situations, F cells can support themselves without M cells. This is a form of fibrosis defined as 'cold fibrosis' by Adler et al, whereas fibrosis with both F and M cells was defined as 'hot fibrosis'.

To model the effect of senescent cells, we note that senescent cells secrete multiple pro-inflammatory factors in the SASP such as IL1, IL6, IL8, and TNF. This can have several effects, each of which can push a given transient injury, which would normally be healed, across the separatrix, causing fibrosis.

These effects are shown in Figure S11: (A) SASP can increase the duration of initial influx of M cells, (B) SASP can increase the number of M cells that flow into the tissue by means of cytokines such as IL1 and IL6, (C) Senescent cells can cause chronic inflammation that increases the initial number of M and/or F cells in the tissue, and (D) SASP can affect the secretion rates of growth factors by M and F cells, moving the separatrix and reducing the region in which the system flows towards the healing state (the basin of attraction of the healing state, shown in gray in Figure S11).

The size of these effects can be effectively described by a parameter ϕ. Once ϕ exceeds a threshold, an injury that would have healed instead causes fibrosis. Micro-injuries associated with the function of organs, with excess lipids as in obesity, blood vessel damage in hypertension, or with toxins such as alcohol in the liver, cause local (focal) fibrosis. As senescent cell levels rise with age, fibrosis becomes more and more likely and occurs in larger and larger parts of these organs. This leads to progressive decline in tissue function with age.

Individuals susceptible to fibrotic diseases by this mechanism are predicted to have genetic and environmental factors that increase the rate of injury in a given tissue. An example is liver cirrhosis, which has risk factors such as viral infection, alcohol and obesity (fatty liver disease) (Schuppan & Afdhal 2008; Karageorgos et al. 2017). An additional example includes some forms of kidney disease in which podocytes are damaged, leading in some cases to end stage kidney disease (ESRD) (Bryer & Susztak 2018; Lin & Susztak 2016).

Indeed, the present model explains the incidence of various fibrotic diseases well (see Fig 2A for end stage kidney disease and liver cirrhosis).

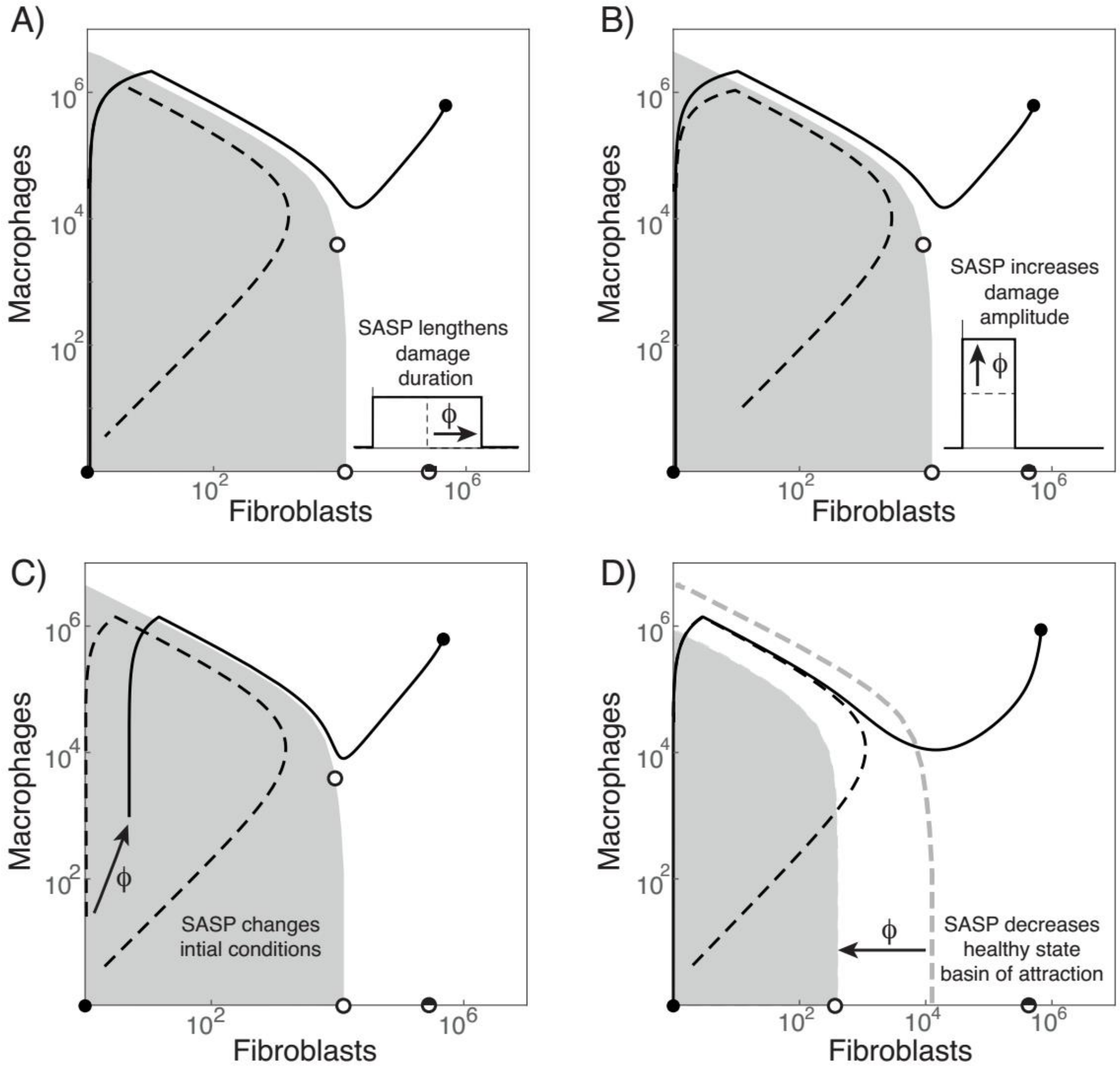

Figure S11: **Senescent cells secrete pro-inflammatory factors that increase the range of injuries that lead to fibrosis in the Adler et al. 2019 model.** SASP secreted by senescent cells can (A) increase the duration of initial influx of macrophages, M, (B) increase the number of M cells that flow into the tissue by means of cytokines such as IL1 and IL6, (C) cause chronic inflammation that increases the initial number of M and/or F cells in the tissue, and (D) affect the secretion rates of growth factors by M and F cells, moving the separatrix and the region in which the system will flow towards the healing state (the basin of attraction of the healing state, shown in gray). The size of these effects is the physiological parameter ϕ. Dashed lines are trajectories of an injury that heals in which M and F cell numbers first rise and then fall, black lines are trajectories that go to fibrosis due to the effects of SASP.

Section 10: Age of maximum incidence in the model rises with disease threshold Xc.

We used the two-parameter model to compute the age of maximum disease incidence as a function of disease thresholds X_c for several values of susceptibility s. We find that the age of maximum incidence rises approximately linearly with X_c, until X_c approaches $X_{death} = 17$. For low susceptibility, *s<0.2*, which applies to almost all of the diseases, this curve is approximately independent on s.

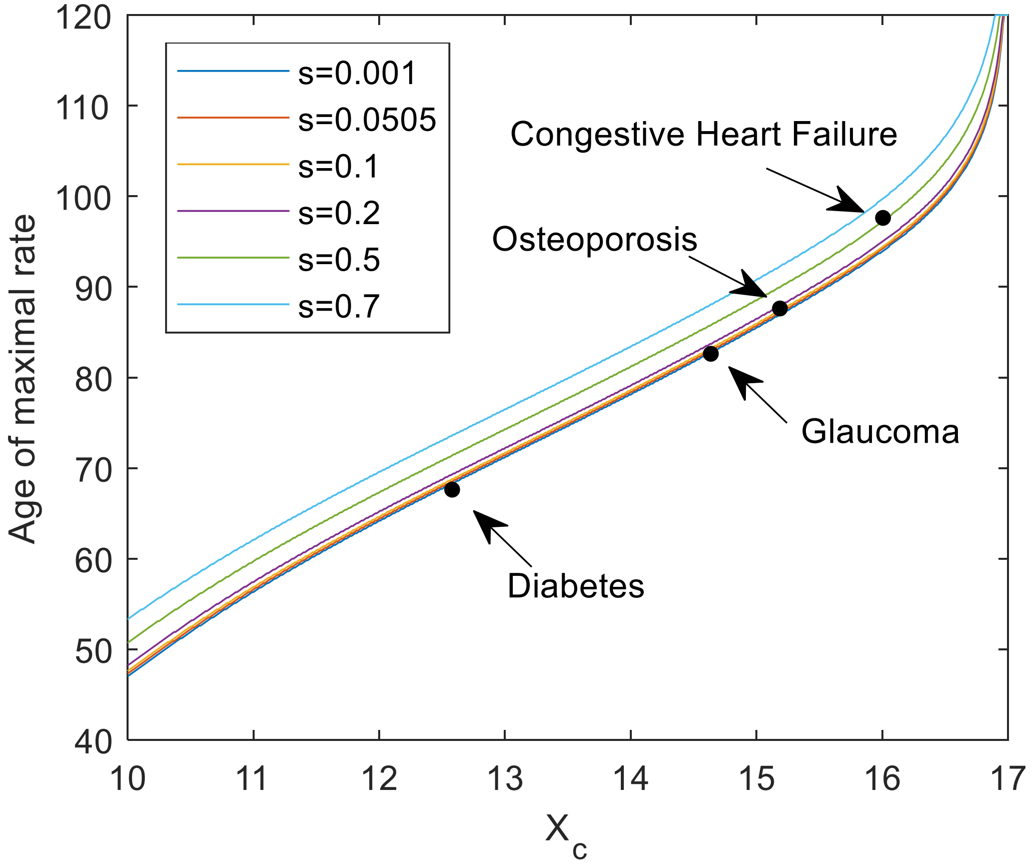

Figure S12: The age of maximal incidence in the model rises approximately linearly with X_c, until X_c comes close to $X_{death} = 17$, when the age of maximal incidence rises sharply. When the susceptibility parameter s is small (s<0.2) the age of maximal rate is almost independent of s. Age of maximal incidence for several diseases from the Clalit dataset is indicated in black dots.

Adler M, Mayo A, Zhou X, Franklin R, Meizlish M, Medzhitov R, Kallenberger S & Alon U (2019) Principles of Cell Circuits for Tissue Repair and Fibrosis. *bioRxiv*, 710012.

Adler M, Mayo A, Zhou X, Franklin RA, Jacox JB, Medzhitov R & Alon U (2018) Endocytosis as a stabilizing mechanism for tissue homeostasis. *Proc. Natl. Acad. Sci. U. S. A.* 115, E1926–E1935.

Bryer JS & Susztak K (2018) Screening Drugs for Kidney Disease: Targeting the Podocyte. Available at: https://doi.org/10.1016/j.chembiol.2018.01.018 [Accessed December 9, 2019].

Chen MJ, Whiteley JP, Please CP, Schwab A, Ehlicke F, Waters SL & Byrne HM (2018) Inducing chondrogenesis in MSC/chondrocyte co-cultures using exogenous TGF-β: a mathematical model. *J. Theor. Biol.* 439, 1–13. Available at: http://www.ncbi.nlm.nih.gov/pubmed/29203122 [Accessed October 31, 2018].

Coppé J-P, Desprez P-Y, Krtolica A & Campisi J (2010) The senescence-associated secretory phenotype: the dark side of tumor suppression. *Annu. Rev. Pathol.* 5, 99–118. Available at: http://www.ncbi.nlm.nih.gov/pubmed/20078217 [Accessed May 26, 2019].

Jiang Y & Tuan RS (2015) Origin and function of cartilage stem/progenitor cells in osteoarthritis. *Nat. Rev. Rheumatol.* 11, 206–212.

Karageorgos SA, Stratakou S, Koulentaki M, Voumvouraki A, Mantaka A, Samonakis D, Notas G & Kouroumalis EA (2017) Long-term change in incidence and risk factors of cirrhosis and hepatocellular carcinoma in crete, Greece: A 25-year study. *Ann. Gastroenterol.* 30, 357–363.

Komarova NL (2013) Principles of Regulation of Self-Renewing Cell Lineages S. R. Proulx, ed. *PLoS One* 8, e72847. Available at: http://www.ncbi.nlm.nih.gov/pubmed/24019882 [Accessed October 31, 2018].

Lander AD, Gokoffski KK, Wan FYM, Nie Q & Calof AL (2009) Cell Lineages and the Logic of Proliferative Control C. F. Stevens, ed. *PLoS Biol.* 7, e1000015. Available at: http://dx.plos.org/10.1371/journal.pbio.1000015 [Accessed April 17, 2018].

Lin JS & Susztak K (2016) Podocytes: the Weakest Link in Diabetic Kidney Disease? *Curr. Diab. Rep.* 16.

McCulloch K, Litherland GJ & Rai TS (2017) Cellular senescence in osteoarthritis pathology. *Aging Cell* 16, 210–218. Available at: http://www.ncbi.nlm.nih.gov/pubmed/28124466 [Accessed May 26, 2019].

Newman SJ (2018) Errors as a primary cause of late-life mortality deceleration and plateaus U. Dirnagl, ed. *PLOS Biol.* 16, e2006776. Available at: https://dx.plos.org/10.1371/journal.pbio.2006776 [Accessed November 3, 2020].

Oliveria SA, Felson DT, Reed JI, Cirillo PA & Walker AM (1995) Incidence of symptomatic hand, hip, and knee osteoarthritis among patients in a health maintenance organization. *Arthritis Rheum.* 38, 1134–41. Available at: http://www.ncbi.nlm.nih.gov/pubmed/7639811 [Accessed October 31, 2018].

Reyes C, Leyland KM, Peat G, Cooper C, Arden NK & Prieto-Alhambra D (2016) Association Between Overweight and Obesity and Risk of Clinically Diagnosed Knee, Hip, and Hand Osteoarthritis: A Population-Based Cohort Study. *Arthritis Rheumatol.* 68, 1869–1875.

Schuppan D & Afdhal NH (2008) Liver cirrhosis. *Lancet* 371, 838–851.

Yang J, Axelrod DE & Komarova NL (2017) Determining the control networks regulating stem cell lineages in colonic crypts. *J. Theor. Biol.* 429, 190–203. Available at: http://www.ncbi.nlm.nih.gov/pubmed/28669884 [Accessed October 31, 2018].

Youssef A, Aboalola D & Han VKM (2017) The roles of insulin-like growth factors in mesenchymal stem cell niche. *Stem Cells Int.* 2017, 1–12. Available at: https://www.hindawi.com/journals/sci/2017/9453108/ [Accessed January 8, 2019].

Zhou X, Franklin RA, Adler M, Jacox JB, Bailis W, Shyer JA, Flavell RA, Mayo A, Alon U & Medzhitov R (2018) Circuit Design Features of a Stable Two-Cell System. *Cell* 172, 744–757.e17.
